# Aberrant plasma MMP and TIMP dynamics in Schistosoma - Immune reconstitution inflammatory syndrome (IRIS)

**DOI:** 10.1371/journal.pntd.0006710

**Published:** 2018-08-08

**Authors:** Odin Goovaerts, Pauline N. M. Mwinzi, Erick M. O. Muok, Ann Ceulemans, Robert Colebunders, Luc Kestens

**Affiliations:** 1 Department of Biomedical Sciences, Institute of Tropical Medicine, Antwerp, Belgium; 2 Neglected Tropical Diseases Unit, Centre for Global Health Research, Kenya Medial Institute, Kisumu, Kenya; 3 Epidemiology for Global Health Institute, University of Antwerp, Antwerp, Belgium; Weill Cornell Medical College, UNITED STATES

## Abstract

**Background:**

Among the different faces of immune reconstitution inflammatory syndrome (IRIS) developing in HIV-patients, no clinical definition has been reported for Schistosomiasis-IRIS (Schisto-IRIS). Although Schisto-IRIS remains largely uninvestigated, matrix metalloproteinases (MMP) and tissue inhibitors of metalloproteinases (TIMP) have previously been associated with *S*. *mansoni* infection and tuberculosis-IRIS. Here, we aimed to investigate the relevance of these markers in Schisto-IRIS.

**Methodology:**

Patients were diagnosed with IRIS related to *S*. *mansoni* within a cohort of patients with Schistosomiasis-HIV co-infection, using a clinical working definition of Schisto-IRIS. We compared 9 patients who developed Schisto-IRIS to 9 Schisto^+^HIV^+^ controls who did not, and 9 Schisto^-^HIV^+^ controls. Plasma levels of MMP-1, MMP-7, MMP-10, TIMP-1, TIMP-2, sCD14, intestinal fatty-acid binding protein, C-reactive protein, and 8 anti-nuclear antibodies (ANA) were analyzed prior to and during 3 months of ART.

**Principal findings:**

Although no differences were observed for MMP-1 and -7, MMP-10 levels decreased significantly in Schisto^+^HIV^+^ controls during 3 months of ART (p = 0.005) while persisting in Schisto-IRIS patients at significantly higher levels compared to Schisto^-^HIV^+^ controls (p≤0.030). In contrast TIMP-1 levels only decreased significantly in Schisto-IRIS patients (p = 0.012), while TIMP-2 levels were lower compared to Schisto^+^HIV^+^ controls at 2 weeks (p = 0.007), 1 month (p = 0.005) and 3 months (p = 0.031) of ART. Five out of 8 ANAs studied decreased significantly in Schisto-IRIS patients after 1 month of ART(p≤0.039), whereas only 1 ANA decreased for Schisto^+^HIV^+^ controls (p = 0.027).

**Conclusions/Significance:**

In this study, we propose a working definition for the diagnosis of Schisto-IRIS in resource limited settings. We report persistent plasma levels of MMP-10, along with a more pronounced decrease in TIMP-1 and ANA-levels, and low levels of TIMP-2 during 3 months of ART. Corresponding to the clinical symptoms, these data suggest that Schisto-IRIS is marked by unbalanced MMP/TIMP dynamics which favor inflammation.

## Author summary

A subset of HIV-patients starting antiretroviral therapy are at risk of developing immune-driven worsening symptoms of a previously treated opportunistic infection. This paradoxical immune reconstitution inflammatory syndrome (IRIS) has been abundantly described in common co-infections such as *M*. *tuberculosis* (TB-IRIS), whereas IRIS associated with *Schistosoma mansoni* (Schisto-IRIS) is less well studied. Nonetheless, HIV and *S*. *mansoni* are highly co-endemic in Sub-Saharan Africa and the considerable clinical burden of Schisto-IRIS in the field should not be underestimated. Moreover, no clinical definition exists to help diagnose this complication. Although little is known about the immune dysregulation in Schisto-IRIS, matrix metalloproteinases (MMPs) and tissue inhibitor of metalloproteinases (TIMPs) have been linked to schistosomiasis and TB-IRIS on account of their role in tissue-destructive inflammation. The current study is nested within a three-month case-control study in schistosomiasis/HIV co-infected fishermen starting ART in Kenya. We propose a clinical working definition for Schisto-IRIS, based on critical evaluation of symptoms developing during ART. Our study now links aberrant dynamics of MMPs and TIMPs to Schisto-IRIS as well. Given the role of MMPs and TIMPs in tissue remodeling and inflammation, our findings suggest that Schisto-IRIS is marked by unbalanced MMP/TIMP dynamics that favor inflammation.

## Introduction

HIV-patients initiating antiretroviral therapy (ART) while dealing with a co-infection are at risk of developing immune reconstitution inflammatory syndrome (IRIS). IRIS is described as a clinical deterioration of HIV patients in the first weeks or months after starting ART, often marked by tissue-destructive inflammation [1–3]. At least three conditions need to be present for an HIV patient to be at risk of IRIS; severe immune suppression, a treated (paradoxical IRIS) -or- undiagnosed (unmasking IRIS) opportunistic infection, and initiation of ART as the trigger. Despite these shared features, IRIS embodies a heterogeneous collection of clinical manifestations [1,2], associated with a plethora of pathogens [3,4].

Among these pathogens, IRIS associated with *Schistosoma mansoni* (Schisto-IRIS) has received only limited attention in research, with few cases reported prior to 2010 [5–7]. Nonetheless, HIV and Schistosomiasis are highly co-endemic in Sub-Saharan Africa [8]. This is especially true in regions with frequent human-water interaction [9,10], e.g. fishing villages along the shores of Lake Victoria. Areas such as these could form focal-points of IRIS development, as demonstrated previously in a Kenyan cohort with ~36% of Schistosomiasis-HIV patients on ART developing worsening schistosomiasis symptoms consistent with IRIS [11]. Thus, while perhaps not as prevalent as IRIS associated with tuberculosis (TB-IRIS) [3,12], the clinical burden of Schisto-IRIS in the field should not be underestimated.

Little is known on the immune dysregulation in patients who develop Schisto-IRIS, nor has the relevance of findings in TB-IRIS for Schisto-IRIS been studied before. Most findings in TB-IRIS can be directly linked to an acute inflammatory response to TB-antigens, coinciding with monocyte activation and a cytokine storm [13–15]. Elevated levels of matrix metalloproteinases (MMPs) have also been reported, with MMP-1, -3, -7, -8, and -10 being of particular interest [16–18]. MMPs hydrolyze various components of the extracellular matrix, allowing classification as collagenases (e.g. MMP-1), gelatinases (e.g. MMP-2), matrilysins (e.g. MMP-7) or stromelysins (e.g. MMP-10) [16]. Along with tissue inhibitors of metalloproteinases (TIMPs), MMPs are functionally involved in inflammation, granuloma formation and tissue remodeling, thus illustrating their involvement in TB lung pathology and symptoms seen in TB-IRIS [17,18]. Cells of the innate immune system have the capacity to produce MMPs and TIMPs [19,20], further suggesting a major contribution of the innate immune system to TB-IRIS development.

In schistosomiasis, MMP-1 and -2 and TIMP-1 and -2 have been linked to active periovular granulomas in humans [21], whereas increased levels of MMP-8 and -10, and TIMP-1 and -2 were observed in mice [22]. Conversely, mice treated with praziquantel (PZQ) showed a decrease in MMP and TIMP levels, although MMP-10 levels increased consistent with ongoing resorption of fibrous tissue [23]. PZQ treatment has been reported to influence schistosome-specific immune responses [24,25]. Indeed, a pro-inflammatory shift in schistosome-specific cytokine production has been observed in patients treated with PZQ, which may contribute to resistance to re-infection [26]. Moreover, one study previously described an inverse relationship between *S*. *haematobium* infection intensity and anti-nuclear antibody (ANA) levels, which rise upon PZQ treatment [27]. Together, these findings highlight protective, but potentially pathogenic immune responses to schistosome antigens following PZQ treatment.

Drawing parallels with TB-IRIS, we hypothesized that Schisto-IRIS is characterized by an over-representation of pro-inflammatory factors after PZQ treatment that might otherwise improve resistance to re-infection. Schisto-IRIS could thus be associated with; increased plasma levels of MMPs or ANAs; decreased plasma levels of TIMPs; and increased monocyte activation (measured by soluble CD14). In addition, we previously reported lower levels of intestinal fatty-acid binding protein (I-FABP) in TB-IRIS patients [13]. Since I-FABP is used as a marker for damage to the intestinal epithelium, we further hypothesized that persisting levels of I-FABP could be associated with intestinal symptoms associated with Schisto-IRIS. Using samples collected from Schistosoma mansoni-HIV co-infected fishermen starting ART in Kenya [11], we conducted a nested 3-month case-control analysis of plasma MMPs, TIMPs, ANAs, sCD14, and I-FABP among those who developed Schisto-IRIS.

## Methods

### Study population

**Fig 1:**
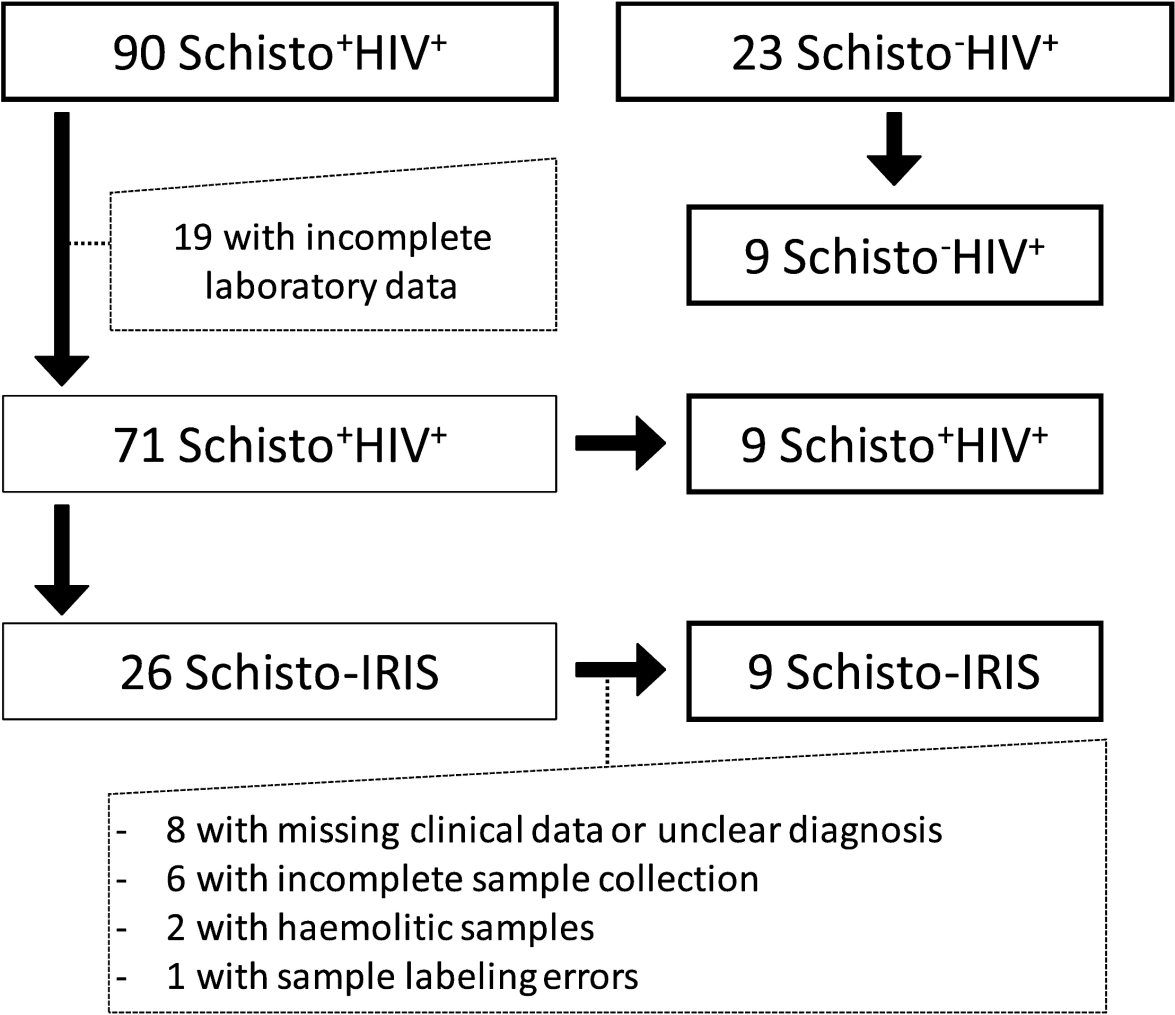
Flow chart of study population. Chart shows the selection of patients for this case-control study, nested within a previous prospective study on HIV-schistosoma co-infection. A number of Schisto-IRIS patients (17/26) were excluded due to sample or data issues, resulting in 9 patients selected with clinical data and non-haemolitic samples available at each time point. Additional Schisto^+^HIV^+^ (n = 9) and Schisto^-^HIV^+^ (n = 9) patients were randomly selected as controls, except for allowing comparable gender distribution between groups.

**Fig 2:**
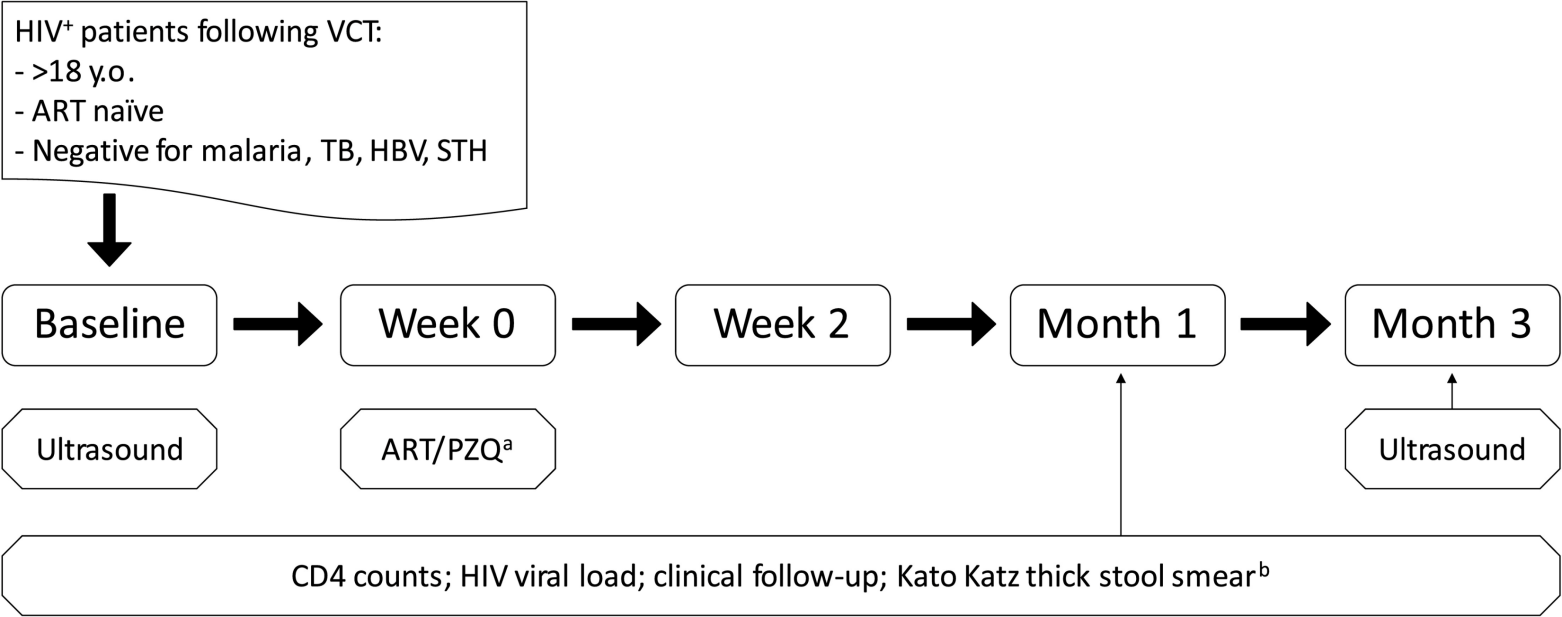
Patient follow-up. Chart shows a time line of patient follow-up during the study. Before enrolment, adult HIV+ patients who were ART naïve and receiving voluntary counseling and testing (VCT) were screened for TB, malaria and hepatitis B virus (HBV), and soil transmitted helminths (STH). Patients then underwent clinical and lab-investigations at baseline (i.e. prior to starting ART and PZQ treatment), and at 2 weeks, 1 month and 3 months after starting ART. Additional screening for signs of *S*. *mansoni* infection by ultrasound were performed at baseline and month 3. ^a^Patients initiated ART and PZQ treatment in close proximity to each other, prior the week 2 visit. Since patients had to be referred to the ART-treatment center, some patients received PZQ before starting ART and some shortly after. ^b^Kato Katz smear was performed at each visit by performing 2 smears on 1 stool sample for quantitative evaluation of *S*. *manoni*, *Ascaris lumbricoides*, *Trichuris trichuria*, hookworm, while *S*. *haematobium* eggs were evaluated by a urine filtration method.

Patients from a prospective case-control study at the fishing community in Uyoma, Rarieda District, Kenya, were studied as described previously [10,11]. The study focused on a group of permanent residents that are occupationally-exposed to water infested with the infective stage of the *Schistosoma mansoni* parasite (S1 Fig). All participating individuals were screened for schistosomiasis and underwent voluntary counseling and testing (VCT) for HIV. All HIV-patients were given lamivudine, stavudine and nevirapine based combination ART shortly after screening, according to Kenya national guidelines at the time. When eggs were identified in stool samples, co-infected patients were also given a single dose of 40mg/kg PZQ treatment according to standard local clinical practice. Seventy-one ART naïve HIV-patients with a history of treated schistosomiasis infection (Schisto^+^HIV^+^) were included in the study, of whom 26 developed Schisto-IRIS. In addition, a group of ART naïve HIV-patients without a history of schistosomiasis infection (Schisto^-^HIV^+^) were recruited as controls (Fig 1). Patients were followed up for 3 months and blood-plasma was collected prior to initiating ART (baseline), at 2 weeks, at 1 and 3 months after starting ART. ART adherence and efficacy were monitored by oral questioning, CD4 counts and viral load (VL) measurements at each time point (Fig 2). In the current study, only patients with available clinical data were included, who had samples of sufficient quality available at all 4 time points to allow longitudinal analysis.

### Diagnostic criteria

Diagnosis of schistosomiasis was performed at baseline and all subsequent time points by Kato Katz thick stool smear as described previously [28]. At each timepoint, 1 stool sample was taken, and 2 smears were analyzed by experienced lab technicians. *S*. *Haematobium* infection was excluded using a parallel urine filtration method [29]. Patients were further excluded when presenting with eggs belonging to other helminth infections; *Ascaris lumbricoides*, *Trichuris trichuria* and hookworm. Other exclusion criteria included; being <18 years of age and having any of the most common co-infections such as malaria, tuberculosis or hepatitis B, respectively detected by microscopy, skin test or serology.

For the purpose of the current study, a working case definition of Schisto-IRIS was refined to match consensus definitions used for the diagnosis of other forms of IRIS (Table 1) [2,30]. Antecedent requirements for paradoxical Schisto-IRIS were; diagnosis of *S*. *mansoni*, followed by successful PZQ treatment in close proximity to ART initiation (and eggs were no longer detectable during the next visit) [7]. Based on a clinical Schisto-IRIS description used previously [11], symptoms were re-evaluated according to frequency and relevance, and classified as “major” or “minor” symptoms. Schisto^+^HIV^+^ patients were diagnosed with paradoxical Schisto-IRIS when presenting with at least 2 major, or 1 major and 3 minor re-emerging symptoms of otherwise successfully treated schistosomiasis during ART. Major symptoms include; new or worsening bloody diarrhea, new or worsening hematuria, and new or worsening hepatomegaly (HMG), splenomegaly (SMG), or portal vein enlargement (PVE) within 3 months of starting ART. Minor symptoms include; constitutional symptoms (fever, night sweats), focal inflammation (skin infection, skin rash, swollen glands, mouth sores), algetic symptoms (chest pain, joint pain), watery diarrhea, and new eggs in stool (whose presence could not be explained by other possible causes). Finally, other possible explanations, such as re-infection, other co-infections and treatment failure were excluded. In accordance with previous IRIS studies, we propose to use the term “ART-associated schistosomiasis” to refer to PZQ naïve individuals who start de novo production of schistosome eggs during ART. Diagnosis of unmasking Schisto-IRIS in patients with ART-associated schistosomiasis required; heightened intensity of clinical manifestations and a clinical course consistent with paradoxical Schisto-IRIS once PZQ treatment was initiated (Table 2).

**Table 1:** Working case definition of paradoxical Schistosoma-IRIS with *S*. *mansoni*.

**Antecedent requirements**
*For paradoxical Schisto-IRIS*:
• Diagnosis of *S*. *mansoni* prior to starting ART, confirmed by positive stool-smear.
• Initial response to *S*. *mansoni* treatment, confirmed by elimination of eggs from stool.
**Clinical criteria**
Of the following, at least 2 major, or 1 major + 3 minor criteria are required:
*Major criteria*:
• New or worsening bloody diarrhea
• New or worsening hematuria
• New or worsening hepato-/splenomegaly within 3 months of ART initiation
• New or worsening portal vein enlargement within 3 months of ART initiation
*Minor criteria*:
• New eggs in stool (whose presence could not be explained by other possible causes)
• Abdominal symptoms; watery diarrhea
• Constitutional symptoms; fever, night sweats
• Signs of focal inflammation; skin infection, skin rash, swollen glands, mouth sores
• Algetic symptoms; chest pain, joint pain
**Other explanations to be excluded**
• Re-infection with *S*. *mansoni*
• Presence of other opportunistic infections
• Poor adherence to, or failure of ART
• Praziquantel failure due to re-emerging immature worms

This working case definition of *S*. *mansoni*-associated IRIS was based on a previously used definition of Schisto-IRIS [11] and refined according to accepted consensus definitions used for TB-IRIS [2] and Cryptococcal-IRIS [30].

**Table 2:** Provisional case definition of unmasking Schistosoma-IRIS in patients with ART-associated Schistosomiasis due to *S*. *mansoni*.

**ART-associated Schistosomiasis**
*We propose that ART-associated Schistosomiasis (cases of Schistosomiasis that are diagnosed during ART) could be defined as follows*:
• Patient is not receiving treatment for Schistosomiasis when ART is initiated.
• De novo production of *S*. *mansoni* eggs in stool during ART in individuals who were not producing eggs before.
**Unmasking Schistosoma-IRIS**
We propose that the following could suggest a diagnosis of unmasking Schistosoma-IRIS:
• Patient fulfills the criteria for ART-associated Schistosomiasis.
• Heightened intensity of clinical manifestations, particularly with an inflammatory component present.
• Once Schistosomiasis treatment is initiated, the patient presents with a clinical course that is complicated by a paradoxical reaction consistent with paradoxical Schistosoma-IRIS criteria^a^.

This working case definition of *S*.*mansoni*-associated IRIS was based on a previously used definition of Schisto-IRIS [11], and refined according to accepted consensus definitions used for TB-IRIS [2] and Cryptococcal-IRIS [30].

^a^De novo egg production is not included in diagnosis of unmasking Schisto-IRIS, as it is an antecedent requirement.

### Plasma analysis

Venous blood was drawn into EDTA tubes and plasma was separated from cells by centrifugation and stored at -80°C. Plasma concentrations of 3 MMPs and 2 TIMPs were determined in duplicate using Bio-Plex human MMP and TIMP assay kits (Bio-Rad Laboratories NV-SA, Nazareth, Belgium) according to the manufacturer’s instructions. We thus measured plasma levels of MMP-1, -7, -10 and TIMP-1 & -2. The Bio-Plex array reader and Manager 5.0 software were used to analyse concentrations using a weighted five-parameter logistic curve-fitting method. In addition, plasma concentrations of sCD14 and intestinal fatty-acid-binding protein (I-FABP) were determined by ELISA (HyCult biotechnology BV, Uden, The Netherlands), and C-reactive protein (CRP) was determined using VITROS Chemistry Products CRP Slides (Ortho-Clinical Diagnostics, NY, USA). In addition, a semi-quantitive determination of 8 ANAs (directed against; Smith antigen (Sm), U1 small nuclear ribonucleoprotein (U1 snRNP), snRNP/Sm complex, Sjögren’s-syndrome-related antigen A & B (SS-A & SS-B), topoisomerase I (Scl-70), centromere protein B (CenpB) and Jo-1) was performed by ELISA using index values, calculated by ratio of sample to cut-off calibrator (AESKU.DIAGNOSTICS GmbH & Co., Wendelsheim, Germany).

### Ethical considerations

The study was approved by the Scientific Steering Committee (SSC number 1763) and Ethical Review Committee (ERC) at the Kenya Medical Research Institute (KEMRI) and written informed consent was obtained from all study participants. The use of plasma samples in the current study was approved by the institutional review board of the Institute of Tropical Medicine of Antwerp.

### Statistical analysis

Statistics were performed using SPSS software (version 17.0) or GraphPad Prism (version 7) with significance level set at p < 0.05. Differences between patient groups were analyzed using Mann-Whitney U tests, or Pearson Chi-square tests for dichotomous values. The significant change over time of variables for each patient group was calculated using the Friedman test (p-values shown in graphs). When Friedman tests showed global significance, Dunn’s multiple comparison post-hoc tests and multiplicity adjusted p-values were used to indicate differences between specific time points, (indicated in graphs by horizontal bars with an asterisk). Significant differences between 2 individual time points (baseline vs. month 1 for ANAs) within groups were determined using a Wilcoxon signed-rank test. Correlations were performed using Spearman's rank-order correlation. Because of the hypothesis driven nature of this study, no other correction for multiple testing was applied [31,32].

## Results

### Study population

**Fig 3:**
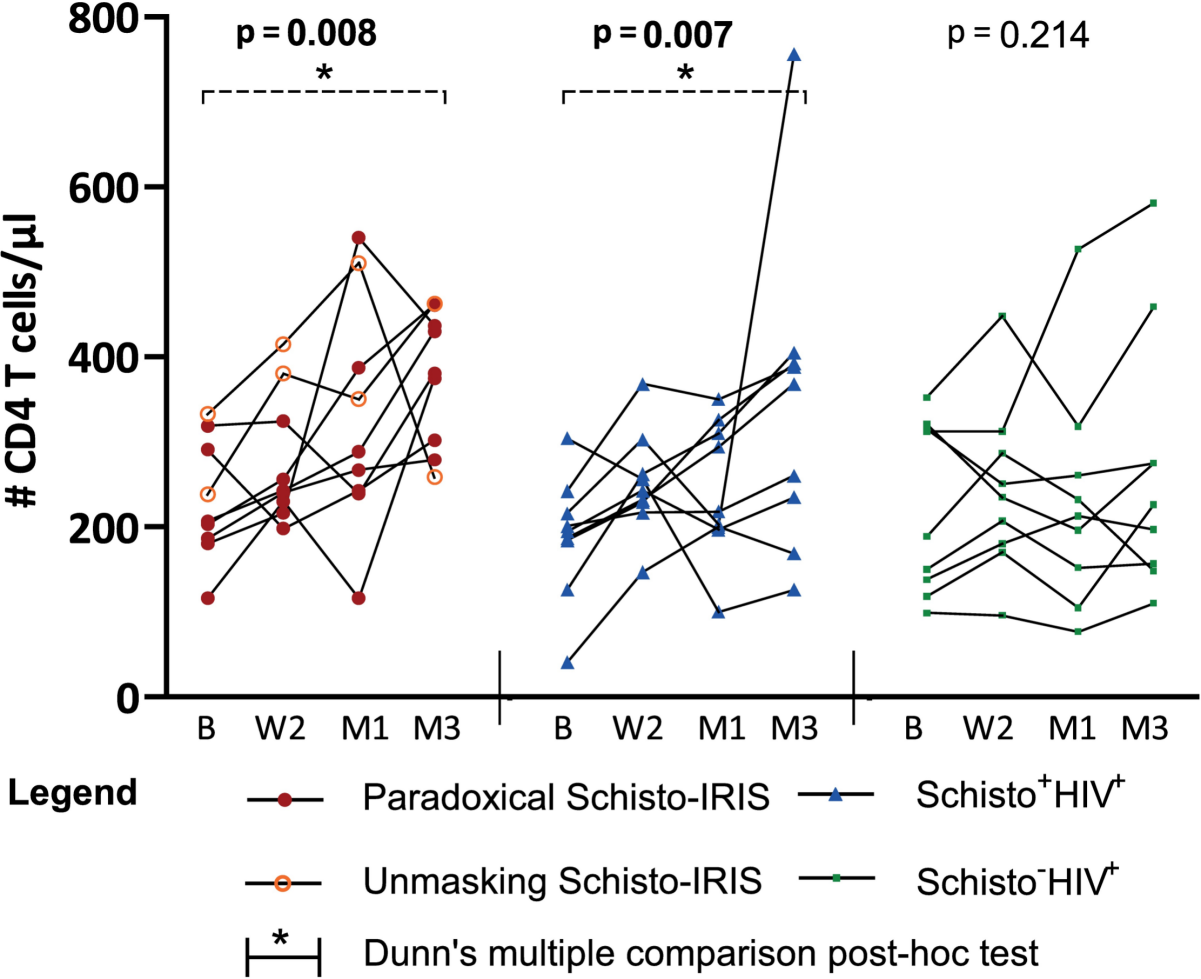
Time analysis of CD4 counts. Graph shows CD4 counts before and during ART in Schisto-IRIS patients (red circles), Schisto^+^HIV^+^ controls (blue triangles) and Schisto^-^HIV^+^ controls (green squares). The significant change over for each patient group was calculated using the Friedman test (p-values shown in graphs), with Dunn’s multiple comparison post-hoc tests to indicate differences between time points (indicated by horizontal bars with an asterisk). B = baseline, W2 = week 2, M1 = month 1, and M3 = month 3.

**Fig 4:**
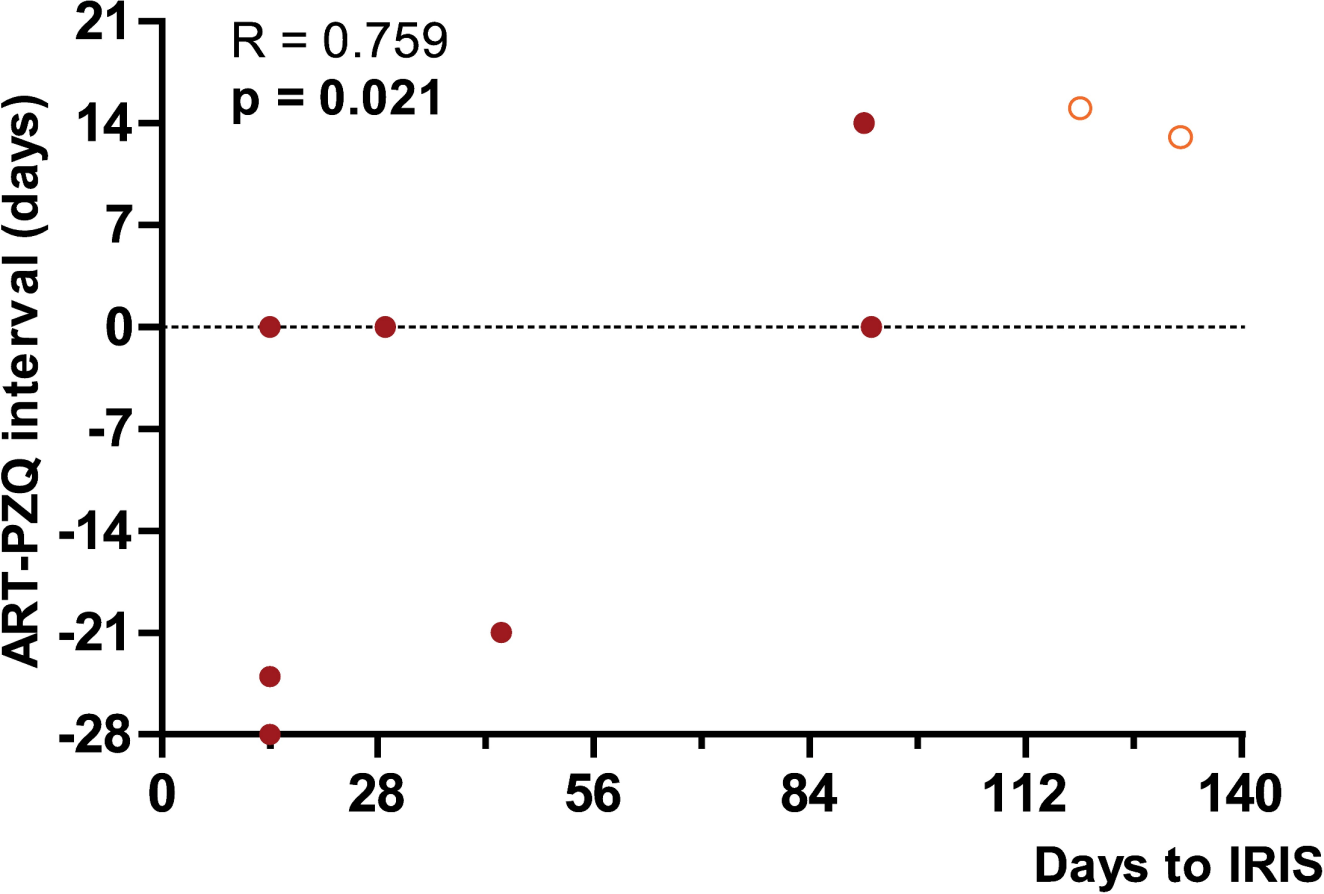
Correlation of treatment interval with onset of IRIS. Graph shows a correlation between ART-PZQ interval and onset of IRIS symptoms (#days since ART initiation), negative values indicate PZQ treatment prior to ART and dotted line represents 0 days interval.

A subset of plasma samples collected within a prospective case-control study at the fishing community in Uyoma, Kenya [11] were selected for further analysis. Plasma samples from a total of 9 Schisto-IRIS patients, 9 Schisto^+^HIV^+^ and 9 Schisto^-^HIV^+^ patients were thus analyzed. All 3 groups did not differ significantly in age, gender or baseline viral load (Table 3). No significant differences could be observed between any of the groups for CD4 counts across all 4 time points, except for Schisto^-^HIV^+^ patients who had lower counts compared to Schisto-IRIS patients after 3 months on ART (p = 0.047). Time analysis showed a significant increase in CD4 counts for Schisto-IRIS patients (p = 0.008) and Schisto^+^HIV^+^ patients (p = 0.007) during 3 months of ART, whereas Schisto-HIV+ participants did not have a significant increase in CD4 counts (p = 0.214) (Fig 3). Dunn’s post-hoc test subsequently highlighted a significant increase from baseline to month 3 in Schisto-IRIS patients (p = 0.004) and Schisto^+^HIV^+^ patients (p = 0.003). Schisto-IRIS patients did not differ from Schisto^+^HIV^+^ patients for baseline egg-count [eggs per gram (EGP)] and EPG were reduced in both groups after PZQ treatment. Due to patients being referred to government facilities for ART, treatment intervals between PZQ and ART varied from patient to patient (Fig 4). Overall, the treatment interval between PZQ and ART did not differ between selected Schisto-IRIS and Schisto^+^HIV^+^ patients (p = 0.287). All patients received PZQ before the standard planned visit at week 2, except for 2 Schisto^+^HIV^+^ patients (who received PZQ 35 and 81 days after ART initiation).

**Table 3:** Clinical characteristics of study population.

	Schisto-IRIS _(n = 9)_	Schisto^+^HIV^+^_(n = 9)_	Schisto^-^HIV^+^_(n = 9)_	p-value^a^		
	(A)	(B)	(C)	(A) vs. (B)	(A) vs. (C)	(B) vs. (C)
**Baseline only characteristics**						
Male (n) (%)	6 (67%)	6 (67%)	5 (56%)	1.000^b^	0.629^b^	0.629^b^
Age (Years)	34 (29–40)	34 (32–35)	35 (32–40)	0.790	0.965	0.658
Viral load^c^	5.0 (4.4–5.6)	3.2 (3.1–5.7)	4.1 (2.8–5.5)	0.201	0.175	0.519
**Follow up characteristics**						
# CD4 cells/μl						
*Baseline*	207 (186–291)	195 (184–216)	189 (138–315)	0.354	0.757	0.627
*Week 2*	243 (230–324)	243 (229–262)	235 (180–287)	0.691	0.354	0.691
*Month 1*	288 (243–387)	218 (200–310)	213 (152–261)	0.157	0.070	0.508
*Month 3*	381 (302–437)	368 (235–392)	226 (157–275)	0.270	**0.047**	0.354
# Eggs/gram						
*Baseline*	12 (12–60)	48 (24–60)	0 (0–0)	0.154	**0.002**	**<0.001**
*Week 2*	0 (0–6)	0 (0–0)	0 (0–0)	0.700	0.169	0.285
*Month 1*	0 (0–0)	0 (0–0)	0 (0–0)	1.000	0.285	0.317
*Month 3*	0 (0–12)	0 (0–0)	0 (0–0)	0.271	0.067	0.289

Data are represented as median and interquartile range unless stated otherwise.

^a^Mann-Whitney U tests were used to calculate significant differences between Schisto-IRIS (A), Schisto^+^HIV^+^ (B), and Schisto^-^HIV^+^ (C) patients.

^b^Pearson Chi-square test.

^c^ Baseline viral loads (Log copies/ml) were available for 6 Schisto-IRIS, 5 Schisto^+^HIV^+^ and 9 Schisto^-^HIV^+^ patients. The level of significance was set to P < 0.05 for all tests.

### Symptoms and onset of Schisto-IRIS

All co-infected patients reported in this study experienced clinical signs consistent with *S*. *mansoni* infection prior to starting PZQ and ART including; hepato-/splenomegaly, bloody/watery diarrhea, abdominal pains, etc. (S1 Table). Patients were retrospectively classified as Schisto-IRIS or Schisto^+^HIV^+^ patients, according to symptoms presented during ART. Both groups showed similar distribution of abdominal symptoms at baseline, while neither group experienced PVE at this time. SMG at baseline was diagnosed in 6/9 (67%) Schisto-IRIS patients but only in 1/9 (11%) Schisto^+^HIV^+^ patients, though 2/9 (22%) Schisto^+^HIV^+^ patients experienced HMG. Following PZQ/ART treatment, EPG declined in all patients. Symptoms subsided within 3 months of ART in patients who were not diagnosed with IRIS. In contrast, 6/9 (67%) Schisto-IRIS patients developed new or worsening bloody diarrhea, 3/9 (33%) developed new HMG or SMG and 5/9 (56%) developed new PVE (Table 4). All Schisto-IRIS patients developed at least 3 minor symptoms during ART, except for one who developed 2 (in addition to 2 major symptoms). This included 7 patients with paradoxical IRIS and 2 patients with unmasking IRIS, whom did not differ in baseline characteristics or CD4 count. Unmasking Schisto-IRIS patients showed zero EPG prior to ART, but experienced de novo egg production at 2 weeks after starting ART. These patients initiated PZQ treatment only after egg production was diagnosed (at 13 and 15 days post ART). Since patients were followed up at pre-determined visits, IRIS was diagnosed at the closest visit. The median time to IRIS diagnosis was 29 days (IQR 14–91) for paradoxical Schisto-IRIS, and 126 days (IQR 119–132) for unmasking Schisto-IRIS (p = 0.053). We observed a significant correlation between treatment interval and time to IRIS diagnosis (R = 0.759; p = 0.021) (Fig 4).

**Table 4:** Description of symptoms in Schisto-IRIS patients.

#	PZQ-ART (days)^a^	IRIS (days)	Pre-ART	Symptoms during 3 months after starting ART
1 (P)	-28	14	abdominal pain, watery diarrhea	Developed bloody diarrhea & skin infection at week 2. Later developed new SMG and minor symptoms (watery diarrhea and night sweats).
2 (P)	0	14	SMG	Developed bloody diarrhea at week 2. Later developed de novo eggs and other minor symptoms (watery diarrhea, fever, and mouth sores).
3 (P)	-24	14	abdominal distension	Developed persistent watery diarrhea at week 2. Later developed new portal vein enlargement, de novo eggs and other minor symptoms (skin infection, and fever).
4 (P)	0	29	SMG abdominal pain, skin infection	Developed bloody diarrhea at month 1. Later developed de novo eggs and other minor symptoms (watery diarrhea, headache, night sweats).
5 (P)	-21	44	SMG, hematuria abdominal pain, fever	Experienced worsening hematuria & fever. Developed watery diarrhea at month 1 and was later diagnosed with new portal vein enlargement and de novo eggs.
6 (P)	14	91	SMG, bloody diarrhea, abdominal pain	Bloody diarrhea persisted until week 2. Was diagnosed with new portal vein enlargement and new hepato-(spleno)megaly at month 3.
7 (P)	0	92	SMG, bloody diarrhea, abdominal pain, swollen glands	Experienced worsening bloody diarrhea. Was diagnosed with new portal vein enlargement at month 3, and minor symptoms (fever and swollen glands).
8 (U)	13	90	SMG, skin infection	Unmasking de novo egg production at week 2, with skin infection and fever. Developed new HMG and portal vein enlargement at month 3.
9 (U)	15	119	abdominal distension, headache	Unmasking de novo egg production at week 2. Developed bloody diarrhea at month 3, with minor symptoms (chest- & joint pain and fever).

Individual patients are numbered and represented as paradoxical (P, with detectable S. mansoni EPG pre-ART) or unmasking (U, with no detectable EPG pre-ART) Schisto-IRIS patients. IRIS (days) represents the number of days between starting ART and first documented appearance of symptoms consistent with Schisto-IRIS. Chronic symptoms such as hepato-/spleno-megaly and portal vein enlargement were determined by ultrasound at month 3 and added to the clinical diagnosis of Schisto-IRIS. SMG = splenomegaly, HMG = hepatopegaly.

^a^negative values represent the number of days between PZQ and ART before starting ART, positive values represent the number of days between ART and PZQ after starting ART.

### Plasma levels of matrix metalloproteinases

**Fig 5:**
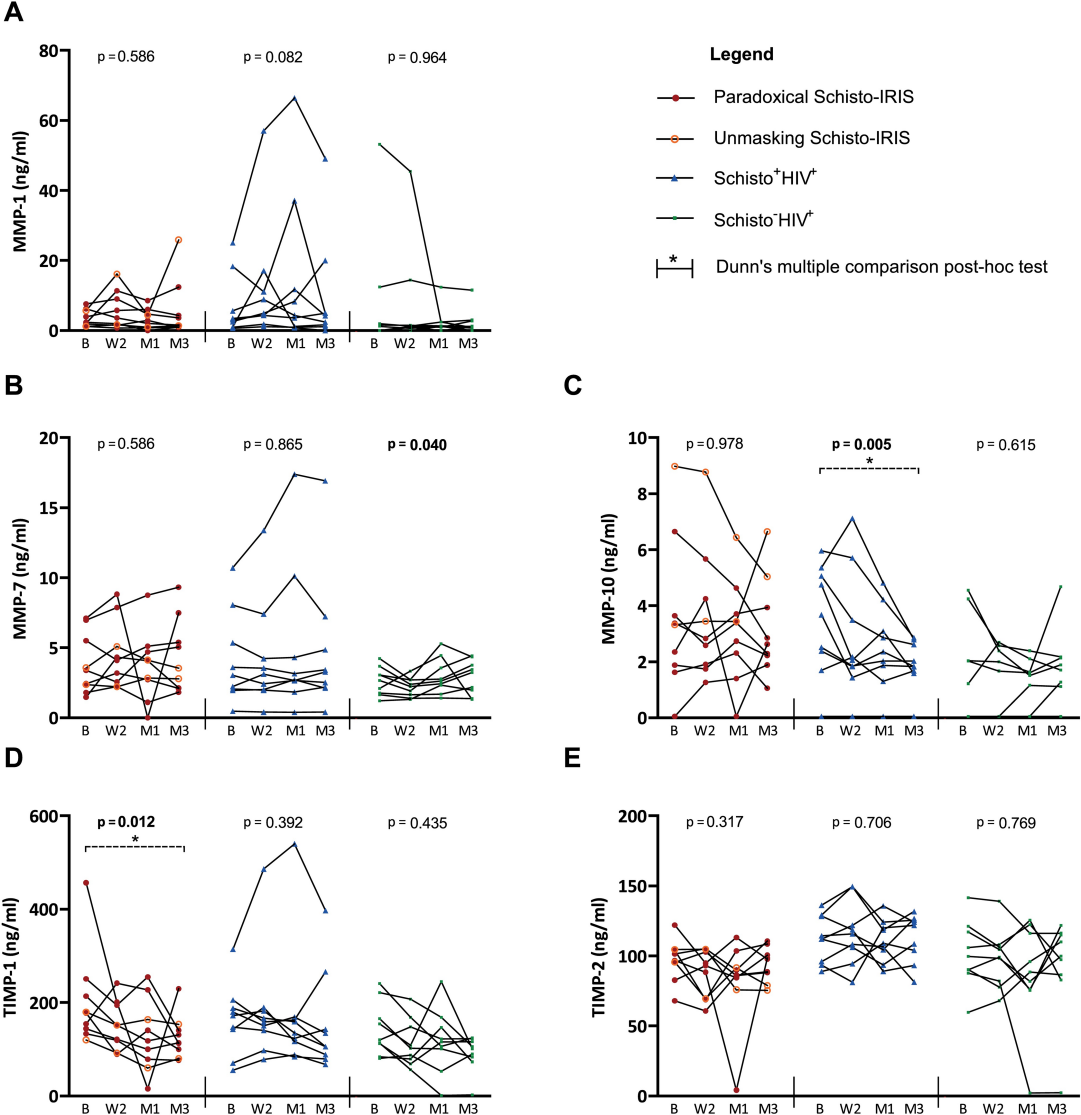
Time analysis of MMPs and TIMPs. Graphs show plasma levels of (A) MMP-1, (B) MMP-7, (C) MMP-10, (D) TIMP-1, and (E) TIMP-2 before and during ART in Schisto-IRIS patients (red circles), Schisto^+^HIV^+^ controls (blue triangles) and Schisto^-^HIV^+^ controls (green squares). The significant change over time for each patient group was calculated using the Friedman test (p-values shown in graphs), with Dunn’s multiple comparison post-hoc tests to indicate differences between time points (indicated by horizontal bars with an asterisk). B = baseline, W2 = week 2, M1 = month 1, and M3 = month 3.

Increased plasma levels of MMPs have previously been associated with Schistosomiasis, TB infection and TB-IRIS [22,23,33–35]. In order to investigate the role of MMPs in Schisto-IRIS, we evaluated 3 different MMPs classified as either collagenase (MMP-1), matrilysin (MMP-7) or stromelysin (MMP-10) [16]. We thus evaluated plasma MMP levels in 3 patient groups at baseline and after 2 weeks, 1 month and 3 months on ART (Table 5). We could not observe significantly different MMP-1, MMP-7 or MMP-10 levels in Schisto-IRIS patients when directly compared to Schisto^+^HIV^+^ patients. However, Schisto-IRIS patients showed significantly higher MMP-10 levels during ART compared to Schisto^-^HIV^+^ patients (p ≤ 0.030). Conversely, MMP-10 levels were similar between Schisto^+^HIV^+^ and Schisto^-^HIV^+^ patients during ART, except for month 1 (p = 0.041). Subsequent analysis over time revealed a significant overall change in plasma MMP-10 levels for Schisto^+^HIV^+^ patients (p = 0.005), with Dunn’s post-hoc test highlighting a significant decrease from baseline to month 3 specifically (p = 0.006). In contrast, Schisto-IRIS and Schisto^-^HIV^+^ patients showed no significant change in MMP-10 levels (Fig 5A–5C).

**Table 5:** Plasma levels of MMPs & TIMPs during follow-up of 3 patient groups.

	Schisto-IRIS _(n = 9)_ (A)	Schisto^+^HIV^+^_(n = 9)_ (B)	Schisto^-^HIV^+^_(n = 9)_ (C)	p-value^a^		
				(A) vs. (B)	(A) vs. (C)	(B) vs. (C)
**MMP-1**						
*Baseline*	2.4 (1.9–5.7)	3.1 (1.1–5.6)	1.2 (0.0–2.0)	0.895	0.120	0.169
*Week 2*	3.7 (1.7–9.0)	4.9 (4.4–11.1)	1.3 (0.6–1.5)	0.402	0.102	0.047
*Month 1*	2.9 (0.9–4.7)	4.4 (1.2–11.8)	1.2 (1.0–2.4)	0.270	0.353	0.102
*Month 3*	1.6 (1.0–4.3)	4.2 (1.6–5.0)	1.0 (0.7–2.8)	0.402	0.122	0.084
**MMP-7**						
*Baseline*	3.4 (2.4–5.5)	3.0 (2.1–5.4)	2.7 (1.8–3.1)	0.965	0.270	0.453
*Week 2*	4.1 (2.6–5.1)	3.1 (2.0–4.2)	2.2 (1.7–2.7)	0.354	**0.019**	0.122
*Month 1*	4.1 (2.7–4.7)	2.7 (2.7–4.3)	2.6 (2.3–3.6)	0.825	0.402	0.508
*Month 3*	3.5 (2.1–5.4)	3.3 (2.2–4.9)	3.2 (2.0–3.8)	0.757	0.233	0.508
**MMP-10**						
*Baseline*	3.3 (1.9–3.6)	3.7 (2.4–5.1)	1.2 (0.1–2.0)	0.659	0.118	**0.036**
*Week 2*	2.8 (1.9–4.3)	2.1 (1.8–3.5)	1.7 (0.1–2.4)	0.508	**0.019**	0.196
*Month 1*	3.4 (2.3–3.7)	2.4 (1.9–3.1)	1.5 (0.1–1.6)	0.427	**0.017**	**0.041**
*Month 3*	2.6 (2.2–3.9)	1.8 (1.7–2.6)	1.7 (1.1–2.1)	0.085	**0.030**	0.452
**TIMP-1**						
*Baseline*	177.9 (144.1–213.8)	172.4 (142.9–188.3)	120.4 (112.3–165.8)	0.627	0.102	0.566
*Week 2*	151.5 (119.4–194.7)	159 (140.6–182.3)	102.4 (79.3–148)	0.825	0.102	0.122
*Month 1*	118.1 (78.5–163.9)	135.1 (117.5–162.6)	115.7 (101.1–146.8)	0.508	0.895	0.310
*Month 3*	114.5 (100.4–140.5)	105.7 (88.9–143)	101.3 (83.6–116.2)	0.965	0.171	0.310
**TIMP-2**						
*Baseline*	96.7 (95.5–104.1)	112.1 (96.1–128.5)	99.7 (90.1–117.1)	0.102	0.757	0.310
*Week 2*	93.1 (69.6–102.9)	115.7 (106.3–121.9)	99.1 (82.1–106.5)	**0.007**	0.270	0.070
*Month 1*	86.3 (84.5–91.5)	109.2 (104.3–120)	88.6 (76.3–116.1)	**0.005**	0.757	0.102
*Month 3*	97.7 (88.1–100.5)	121.9 (104–125.6)	99.9 (86.6–114.9)	**0.031**	0.453	0.122

Data are represented as median and interquartile range in ng/ml.

^a^Mann-Whitney U tests were used to calculate significant differences between Schisto-IRIS (A), Schisto^+^HIV^+^ (B), and Schisto^-^HIV^+^ (C) patients. Significant p-values are highlighted in bold. The level of significance was set to P < 0.05.

### Plasma levels of tissue inhibitors of metalloproteinases

We next investigated plasma levels of TIMP-1 and TIMP-2 to evaluate their role in Schisto-IRIS (Table 5). TIMP-1 levels did not differ significantly between the 3 patient groups at any time point. Although TIMP-1 levels declined over time in each group, this change over time only reached significance in Schisto-IRIS patients (p = 0.012, Fig 5D and 5E). Dunn’s post-hoc test subsequently highlighted a significant decline from baseline to month 3 in these patients (p = 0.012). Conversely, Schisto-IRIS patients had markedly lower TIMP-2 levels across the board compared to Schisto^+^HIV^+^ patients, reaching significance at week 2 (p = 0.007), month 1 (p = 0.005) and month 3 (p = 0.031). No differences over time could be observed in TIMP2 levels within the Schisto^+^HIV^+^ group.

In order to evaluate the balance between MMPs and TIMPs, we next analyzed ratios of MMP-1, MMP-7 and MMP-10 to TIMP-1 and TIMP-2 in patients and controls (S2 Fig). The ratio of MMP-10/TIMP-2 decreased over time in Schisto^+^HIV^+^ patients (p = 0.012), whereas the ratio of TIMP-1/TIMP-2 decreased in Schisto-IRIS patients only (p = 0.028).

### Plasma levels of CRP, I-FABP, and sCD14

**Fig 6:**
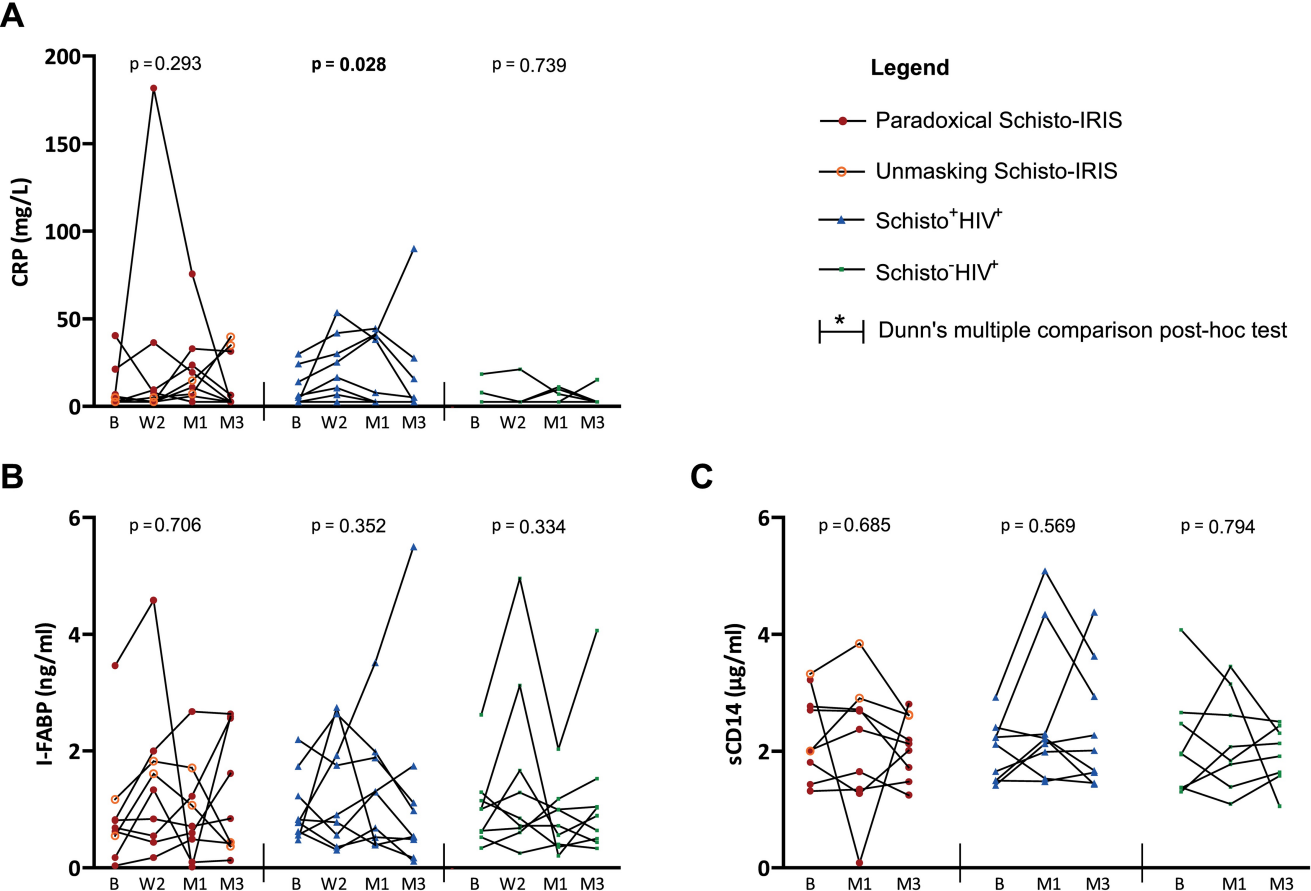
Time analysis of CRP, I-FABP and sCD14. Graphs show plasma levels of (A) CRP, (B) I-FABP and (C) sCD14 before and during ART in Schisto-IRIS patients (red circles), Schisto^+^HIV^+^ controls (blue triangles) and Schisto^-^HIV^+^ controls (green squares). The significant change over time for each patient group was calculated using the Friedman test (p-values shown in graphs), with Dunn’s multiple comparison post-hoc tests to indicate differences between time points when applicable (indicated by horizontal bars with an asterisk). One Schisto^-^HIV^+^ patient did not have sufficient sample volume for CRP determination. B = baseline, W2 = week 2, M1 = month 1, and M3 = month 3.

I-FABP is released into the bloodstream upon damage to the intestinal epithelium, sCD14 is shed upon monocyte activation, and CRP is a well-known acute phase protein. In theory, these markers could therefore be used for monitoring tissue damage and inflammation in Schistosomiasis and/or Schisto-IRIS. We thus evaluated plasma levels of these markers in all 3 patient groups (Table 6 & Fig 6). However, all patients showed comparable I-FABP & sCD14 levels at every time point. No differences were observed for CRP levels between Schisto-IRIS and Schisto^+^HIV^+^ patients either. Compared to Schisto^-^HIV^+^ patients, Schisto-IRIS and Schisto^+^HIV^+^ patients showed higher CRP levels after 1 month (p = 0.021) and 2 weeks (p = 0.012) of ART respectively. Only Schisto^+^HIV^+^ patients showed significant overall variation in CRP over time (p = 0.028), while Dunn’s post-hoc test showed no significance.

**Table 6:** Plasma levels of TIMPs, CRP and I-FABP during follow-up of 3 patient groups.

	Schisto-IRIS _(n = 9)_ (A)	Schisto^+^HIV^+^_(n = 9)_ (B)	Schisto^-^HIV^+^_(n = 9)_ (C)	p-value^a^		
				(A) vs. (B)	(A) vs. (C)	(B) vs. (C)
**CRP(mg/L)**^**b**^						
*Baseline*	5.1 (2.5–6.8)	5.2 (2.5–14)	2.5 (2.5–3.9)	0.890	0.281	0.236
*Week 2*	5.0 (2.5–9.5)	16.6 (6.8–30.1)	2.5 (2.5–2.5)	0.261	0.091	**0.012**
*Month 1*	14.7 (7–23.5)	7.9 (2.5–40.4)	2.5 (2.5–7.7)	0.755	**0.021**	0.211
*Month 3*	2.5 (2.5–31.4)	2.5 (2.5–15.8)	2.5 (2.5–2.5)	0.846	0.136	0.136
**I-FABP (ng/ml)**						
*Baseline*	0.7 (0.5–0.8)	0.8 (0.6–1.2)	1 (0.6–1.2)	0.453	0.508	0.965
*Week 2*	1.3 (0.5–1.8)	0.9 (0.6–1.9)	0.8 (0.7–1.7)	0.965	0.965	0.965
*Month 1*	0.7 (0.5–1.2)	1.3 (0.5–1.9)	0.7 (0.4–1)	0.402	0.825	0.270
*Month 3*	0.8 (0.4–2.6)	0.5 (0.5–1.1)	0.9 (0.5–1)	0.825	0.965	0.757
**sCD14 (**μ**g/ml)** ^**b**^^,^ ^**c**^						
*Baseline*	2.0 (1.6–3.0)	2.1 (1.5–2.3)	2.0 (1.4–2.6)	0.605	0.423	0.815
*Month 1*	2.4 (1.3–2.8)	2.2 (1.8–3.3)	2.0 (1.5–3.0)	0.796	0.963	0.481
*Month 3*	2.1 (1.6–2.6)	2.0 (1.6–3.3)	2.0 (1.6–2.4)	0.931	0.606	0.700

Data are represented as median and interquartile range.

^a^Mann-Whitney U tests were used to calculate significant differences between Schisto-IRIS (A), Schisto^+^HIV^+^ (B), and Schisto^-^HIV^+^ (C) patients.

^**b**^Due to sample limitations, CRP or sCD14 was not determined in 1 Schisto^-^HIV^+^ patient (n = 8).

^**c**^sCD14 was not determined at week 2 for any group. Significant p-values are highlighted in bold. The level of significance was set to P < 0.05.

### Semi-quantitative analysis of auto-antibodies

**Fig 7:**
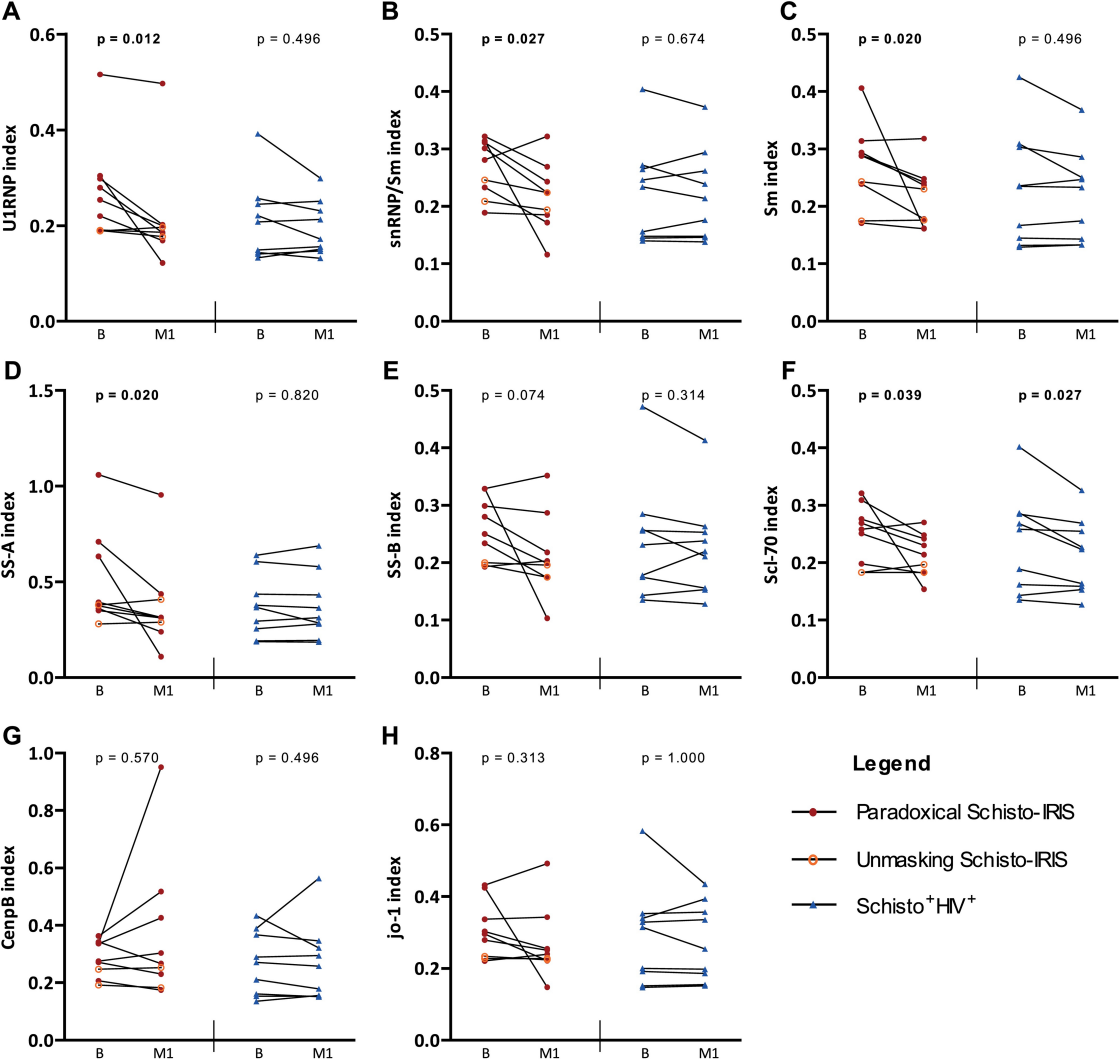
Time analysis of 8 anti-nuclear antibodies. Graphs show semi-quantitative values of plasma levels of ANAs, calculated as a ratio of positive cut-off control (index value). Index values of (A) U1 snRNP, (B) snRNP/Sm complex, (C) Sm, (D) SS-A, (E) SS-B, (F) Scl 70, (G) CenpB and (H) Jo-1 before and during 1 month of ART are shown in Schisto-IRIS patients (red circles) and Schisto^+^HIV^+^ controls (blue triangles). The significant change over time for each patient group was calculated using the Wilcoxon-signed rank test (p-values shown in graphs). B = baseline, M1 = month 1.

Anti-nuclear antibodies are associated with auto-immune diseases and are reported to rise in *S*. *haematobium* patients following PZQ treatment [27]. We thus performed a semi-quantitative analysis of 8 ANAs in Schisto-IRIS and Schisto^+^HIV^+^ patients at baseline and after 1 month of ART (Fig 7). No significant differences could be observed between patient groups at either time point. Nonetheless, Schisto-IRIS showed an overall decrease in plasma levels of 5 ANAs after 1 month of ART; U1-RNP (p = 0.012), SnRNP/Sm (p = 0.027), Sm (p = 0.020), Scl-70 (p = 0.039), SS-A (p = 0.020), SS-B (p = 0.074), CenpB (p = 0.570), Jo-1 (p = 0.313), whereas Schisto^+^HIV^+^ patients only showed decreased levels of Scl-70 (p = 0.027). Comparison of change over time (delta-values calculated by subtracting baseline from month 1) showed a significantly stronger decline of SnRNP/Sm (p = 0.046) and SS-A (p = 0.008) in Schisto-IRIS patients compared to Schisto^+^HIV^+^ patients.

### Correlations with CD4 count, EPG and CRP

Since the number of CD4+ T cells directly influence immunological processes, we next correlated CD4 counts to our observations. Schisto^+^HIV^+^ patients showed a significant negative correlation between CD4 counts and MMP-1, pre-ART (R = -0,683; p = 0.042) and at week 2 (R = -0,717; p = 0.030), whereas Schisto-IRIS and Schisto^-^HIV^+^ patients did not. Next, we evaluated whether excretion of *S*. *mansoni* eggs could be associated with damage to the intestinal epithelium. However, I-FABP levels showed no correlation with EPG at any time point in any group.

To evaluate whether MMP-10 levels could be maintained by inflammatory factors, we then performed a correlation with CRP levels. However, no correlations were observed between CRP and MMP-10 levels at any time point for any group (S3 Fig). We next correlated CRP levels with ANA levels to evaluate a potential link between ANA-levels and systemic inflammation (S4 Fig and S5 Fig). Schisto-IRIS patients showed moderate to strong correlations at baseline between CRP levels and levels of U1-RNP, SS-A, Jo-1, and SS-B (R ≥ 0.669; p ≤ 0.043). Except for U1-RNP, these correlations were preserved at month 1 (R ≥ 0.695, p ≤ 0.043). Schisto^+^HIV^+^ patients showed a similar pattern of correlations at baseline, although CenpB correlated to CRP instead of SS-B (R = 0.783, p = 0.017). At month 1, CRP correlated with SS-A, SS-B, Sm, and CenpB (R ≥ 0.722, p ≤ 0.031) in these patients. Nonetheless, CPR and ANA levels did not show a similar change over time, as no correlation could be observed between delta values (baseline subtracted from month 1) of CRP and any of the ANAs determined. Finally, we evaluated a potential link between the decreasing TIMP-1 and ANA levels in Schisto-IRIS patients (S6 Fig and S7 Fig). However, only Schisto^+^HIV^+^ patients showed correlations between TIMP-1 and 6 of the 8 ANAs at both time points (R ≥ 0.700; p ≤ 0.043).

## Discussion

Among the different faces of IRIS developing in HIV-patients, Schistosomiasis-associated IRIS remains largely uninvestigated despite both infections being highly co-endemic [8]. Unlike clinical definitions used for TB-IRIS [2] and Cryptococcal IRIS [30], no consensus definition for Schisto-IRIS exists. Within a previously described cohort of HIV-patients with Schistosomiasis co-infection, 36.6% developed Schisto-IRIS [11]. Here, we report clinical characteristics of patients who developed Schisto-IRIS related to *S*. *mansoni* and propose a working definition for the diagnosis of paradoxical and unmasking Schisto-IRIS in resource limited settings. In addition, we explored the immunopathogenesis of Schisto-IRIS by measuring plasma levels of 3 MMPs, 2 TIMPs, sCD14, and 8 anti-nuclear antibodies, which were previously suggested to have a role in TB-IRIS and/or schistosomiasis [22,23,27,33–37]. We hypothesized that Schisto-IRIS could be associated with increased plasma levels of MMPs, sCD14, and ANAs; or decreased plasma levels of TIMPs. To that end, we compared plasma levels of these markers during 3 months of ART between Schisto-IRIS patients and Schisto^+^HIV^+^, as well as Schisto^-^HIV^+^ controls. In addition, we explored I-FABP levels as a marker of intestinal damage. We report a significant decline in MMP-10 levels following ART initiation in Schisto^+^HIV^+^ controls, but not in Schisto-IRIS patients. Conversely, plasma levels of TIMP-1 decreased in Schisto-IRIS patients, and TIMP-2 levels were significantly lower shortly after starting ART. In line with our hypothesis, these findings suggest that Schisto-IRIS patients in our cohort experience a MMP/TIMP profile that favors inflammation and tissue damage. Contrary to our hypothesis, plasma levels of ANAs decreased upon ART in Schisto-IRIS patients, possibly reflecting the release of *S*. *mansoni* antigens upon PZQ treatment [27].

The functions ascribed to MMPs range from tissue remodeling and angiogenesis to regulation of immune responses and inflammation [38]. In Schistosomiasis, MMPs regulate the granulomatous response to schistosome-eggs [22]. Upon praziquantel treatment, MMP levels decline in parallel with diminishing inflammatory responses [23]. In line with this, Schistosomiasis-HIV patients in our cohort who did not develop Schisto-IRIS showed a significant decline in MMP-10 levels during ART. Schisto-IRIS patients, however, experienced persistent levels of MMP-10 throughout follow-up. Although we observed no significant differences in MMP-1, -7 or -10 in direct comparison between these 2 groups, Schisto-IRIS patients retained significantly higher MMP-10 levels compared to Schisto^-^HIV^+^ controls, whereas Schisto^+^HIV^+^ controls did not. Overall, these findings indicate that Schisto-IRIS patients do not readily normalize MMP-10 levels within the first months of ART. The spread in data likely masked this effect in direct comparison between groups. Interestingly, MMP-10 gene expression has previously been observed to be paradoxically elevated in praziquantel-treated mice, matching declining collagen gene expression [23]. The persistent levels of MMP-10 in Schisto-IRIS patients observed here could therefore indicate an ongoing immune response to *S*. *mansoni* antigens [39], or a continuous resorption of fibrous tissue surrounding schistosome-eggs [23]. Alternatively, MMP-10 levels could have been maintained by inflammatory factors such as CRP, interferon-gamma, interleukin-6, and/or monocyte activation [40,41], which have previously been associated with TB-IRIS [13,42]. However, we could not observe significant correlations between CRP levels and MMP-10 in our current study. Moreover, no differences were observed for sCD14, which is consistent with our previous observations on TB-IRIS [13].

Counterbalancing the effects of MMPs, TIMPs are natural inhibitors of MMP activity [43]. The balance between MMPs and TIMPs thus influences the level of tissue degradation and inflammation [38]. As expected after praziquantel treatment [23], TIMP-1 levels declined during ART in both Schistosoma-infected groups. However, this decline was much more pronounced in Schisto-IRIS patients. Conversely, TIMP-2 levels remained stable after ART initiation in all groups, but were significantly lower in Schisto-IRIS patients compared to Schisto^+^HIV^+^ patients. These findings suggest that Schisto-IRIS patients have a lowered capacity to compensate for the inflammatory effects of MMP-10 which seem to persist during ART. Interestingly, Schisto^-^HIV^+^ controls showed similarly low levels of TIMP-2 during ART as Schisto-IRIS patients. However, MMP-10 levels were lower still, leading to significantly decreased MMP-10/TIMP-2 ratios at week 2 compared to Schisto-IRIS patients. Although the difference in MMP-10/TIMP-2 ratios between Schisto-IRIS and Schisto^+^HIV^+^ patients did not reach significance, only Schisto^+^HIV^+^ patients showed a significant decrease of this ratio during ART. Overall, these findings suggest diverging TIMP & MMP dynamics in Schistosomiasis-HIV patients who develop IRIS, favoring inflammation and/or tissue damage which becomes clinically apparent with the onset of IRIS symptoms.

All co-infected patients reported in this study experienced clinical signs consistent with *S*. *mansoni* infection prior to starting PZQ and ART [44,45]. Following effective PZQ/ART treatment, most symptoms gradually subsided within 3 months of ART in patients who did not develop IRIS, in line with decreasing levels of MMP-10. Using the working definition proposed in this study, Schisto-IRIS patients could be distinguished from Schisto^+^HIV^+^ controls, as the clinical condition worsened during ART. Compared to baseline, all 9 Schisto-IRIS patients in our study developed new or worsening symptoms of schistosomiasis during this period, despite successful PZQ treatment. In line with persistent MMP-10 levels, a steeper decline in TIMP-1 and consistently low TIMP-2 levels suggest that these patients experience a more vigorous and extended period of tissue damage and/or inflammation. Indeed, the majority of Schisto-IRIS patients developed bloody diarrhea (>50%), new PVE (>50%), and/or new HMG/SMG (>30%) during ART, often accompanied by minor symptoms such as fever, etc. Nonetheless, this did not coincide with altered damage to the intestinal epithelium, as we observed no differences in I-FABP. Overall, our observations on MMP/TIMP dynamics during ART thus correspond to the clinical spectrum of Schisto-IRIS patients as defined here, and may drive or be driven by the ‘major’ symptoms which occurred more rarely in Schisto^+^HIV^+^ controls [46–48]. Nonetheless, the distribution of symptoms among IRIS patients during ART remains somewhat heterogeneous, as is to be expected in IRIS [2,30]. Moreover, the interactions of MMPs and TIMPs are very complex. Consequently, it is difficult to predict the specific roles these factors may have, and what other factors (e.g. cytokines) may be involved. The immune responses and cytokine profiles of Schisto-IRIS patients should therefore be further explored.

Despite the heterogeneity that surrounds IRIS, a high pre-ART antigen burden is commonly recognized as a risk factor, as is a short interval between treatment for the opportunistic infection and ART [49,50]. Although the PZQ-ART interval was similar between our patients and controls, we observed a correlation between PZQ-ART interval and onset of IRIS symptoms. Patients who received PZQ treatment sooner relative to ART (i.e. before ART) showed earlier onset of IRIS symptoms than patients who received PZQ later (i.e. during the first 2 weeks of ART), suggesting a time-dependent association between PZQ treatment, ART initiation, and Schisto-IRIS. Interestingly, studies in HIV-negative patients have demonstrated a rise in pro-inflammatory responses to *S*. *haematobium* [26,51] and *S*. *mansoni* [52,53] antigens within the first months following PZQ treatment. These responses may reflect either the removal of worm-induced immunosuppression or the release of adult worm antigens as a result of PZQ treatment [51]. Although our study did not directly determine antigen levels, we determined relative plasma levels of 8 anti-nuclear antibodies, which have previously been reported to correlate inversely with intensity of *S*. *haematobium* infection and rise during PZQ treatment [27]. In contrast to Schisto^+^HIV^+^ controls, our Schisto-IRIS patients showed a significant decrease in plasma levels of several ANAs after 1 month of ART. Given the inverse relationship of ANA-levels with infection intensity, one could argue that this decrease reflects a sudden release of antigens. Alternatively, a stronger S. *mansoni* specific immune response to these antigens could be present which indirectly downregulates ANA levels. However, this decrease was not mirrored in CRP levels, since no correlation could be observed in change over time (delta value) with ANAs. Considering the time-dependent association with PZQ treatment, it is thus plausible that Schisto-IRIS manifests itself as an aggravated reaction to antigens released by worms that were killed as a result of PZQ treatment. Still, additional studies are needed to fully explore the relationship between ANAs and *S*. *mansoni* antigen loads in Schisto-IRIS.

Although this work is nested in one of the first Schisto-IRIS specific studies to date, our strict selection of patients with complete samples and follow-up data lead to a relatively small population. As we did not include a Schisto^+^HIV^-^ population, our study also cannot provide additional insights in the effects of PZQ without ART. Since this study was focused on *S*. *mansoni* co-infection, the occurrence of IRIS with other species (e.g. *S*. *haematobium*) has also not been investigated. The results reported here should thus inspire larger studies to fully explore MMP profiles in Schisto-IRIS, associated with different Schistosoma species. Our study accounted for re-infection with *S*. *mansoni* by monitoring patients at intervals shorter than 6 weeks. Although exposure between study visits cannot be fully accounted for, all Schisto-IRIS patients developed symptoms before any de novo eggs were documented in stool. We identified onset of IRIS symptoms at the nearest pre-planned visit, with 5 patients showing onset of IRIS symptoms earlier than 45 days on ART, and 4 later than 89 days on ART. Thus, our sample collection did not include IRIS-specific time points, but instead spans the timeframe in which IRIS developed. Nonetheless, as our observations persist across multiple time points, the lack of an IRIS time point did not affect our conclusions. Moreover, no differences could be observed when comparing early- and late-onset IRIS cases for any of the parameters analyzed. Finally, our patient selection included 2 unmasking IRIS patients, making our population more heterogeneous. These patients showed no significant variation compared to paradoxical IRIS patients for any of the clinical variables tested (apart from EPG). However, unmasking Schisto-IRIS patients showed some modest differences in laboratory markers at month 3. Since both cases initiated PZQ treatment after starting ART, this timing could have altered MMP/TIMP dynamics we observed at month 3. Nonetheless, these patients also developed IRIS symptoms at this time point, which likely explains these differences. A larger unmasking Schisto-IRIS population is required to fully explore differences with paradoxical Schisto-IRIS.

In conclusion, we describe characteristics of patients who developed IRIS related to *S*. *mansoni* in one of the first Schisto-IRIS cohorts to date. Therefore, we propose a refined working definition for the diagnosis of paradoxical and unmasking Schisto-IRIS in resource limited settings. Schisto-IRIS patients in our study showed persistent plasma levels of MMP-10, along with a steep decline in TIMP-1 and low levels of TIMP-2 during 3 months of ART. Consistent with the IRIS symptoms reported in the definition, these aberrant MMP and TIMP dynamics suggest the presence of prolonged inflammation and/or tissue damage. Although further research is required, decreasing levels of anti-nuclear antibodies in Schisto-IRIS patients following PZQ/ART may reflect a PZQ-induced release of *S*. *mansoni* antigens, which in turn may drive Schisto-IRIS inflammation. Elucidating the immune pathogenesis behind this complication could lead to treatment strategies for Schisto-IRIS, as well as provide insight in the heterogeneous disease that is IRIS in general.

## Supporting information

S1 Table Summary of symptoms in Schisto-IRIS patients and controls. Prevalence of individual symptoms within selected patients are summarized as number of patients and percentage (n (%)).^a^Two unmasking Schisto-IRIS cases are included, whom had 0 eggs pre-ART and had de novo egg production during ART. All paradoxical Schisto-IRIS patients had detectable eggs in stool pre-ART and 4 (44,4%) of them experienced de novo egg production during follow-up. Table includes both new and persistent symptoms.
(DOCX)

S1 Fig Flow chart of Schisto-IRIS patient follow-up and diagnosis. Figure shows follow-up of HIV patients during ART, with sequential testing for clinical parameters to diagnose schistosomiasis and IRIS.
(TIF)

S2 Fig MMP-10 to TIMP-2 ratio. Figure shows (A) time analysis of MMP-10/TIMP-2 ratio in 3 patient groups using a Friedman test (p-values shown in graphs), with Dunn’s multiple comparison post-hoc tests to indicate differences between time points when applicable (indicated by horizontal bars with an asterisk). (B) comparison of MMP-10/TIMP-2 ratio between groups for each time point using a Mann-whitney U test (p-values and horizontal bars).
(TIF)

S3 Fig Correlation of baseline CRP and MMP-10. Figure shows a correlation within pooled Schisto-IRIS & Schisto^+^HIV^+^ patients between plasma CRP and MMP-10 levels at (A) baseline, (B) week 2, (C) month 1, and (D) month 3. P-values were calculated using a Spearman's rank-order correlation test with significance set to P <0.05.
(TIF)

S4 Fig Correlation of baseline CRP and ANA index. Figure shows a correlation in Schisto-IRIS (red circles) and Schisto^+^HIV^+^ (blue triangles) patients between baseline values for CRP and (A) U1-RNP, (B) snRNP/Sm, (C) Sm, (D) SS-A, (E) SS-B, (F) Scl-70, (G) CenpB, and (H) Jo-1. Each graph represents an individual ANA. P-values were calculated using a Spearman's rank-order correlation test with significance set to P <0.05.
(TIF)

S5 Fig Correlation of month 1CRP and ANA index. Figure shows a correlation in Schisto-IRIS (red circles) and Schisto^+^HIV^+^ (blue triangles) patients between month 1 values for CRP and (A) U1-RNP, (B) snRNP/Sm, (C) Sm, (D) SS-A, (E) SS-B, (F) Scl-70, (G) CenpB, and (H) Jo-1. Each graph represents an individual ANA. P-values were calculated using a Spearman's rank-order correlation test with significance set to P <0.05.
(TIF)

S6 Fig Correlation of baseline TIMP-1 and ANA index. Figure shows a correlation in Schisto-IRIS (red circles) and Schisto^+^HIV^+^ (blue triangles) patients between baseline values for TIMP-1 and (A) U1-RNP, (B) snRNP/Sm, (C) Sm, (D) SS-A, (E) SS-B, (F) Scl-70, (G) CenpB, and (H) Jo-1. Each graph represents an individual ANA. P-values were calculated using a Spearman's rank-order correlation test with significance set to P <0.05.
(TIF)

S7 Fig Correlation of month 1 TIMP-1 and ANA index. Figure shows a correlation in Schisto-IRIS (red circles) and Schisto^+^HIV^+^ (blue triangles) patients between month 1 values for TIMP-1 and (A) U1-RNP, (B) snRNP/Sm, (C) Sm, (D) SS-A, (E) SS-B, (F) Scl-70, (G) CenpB, and (H) Jo-1. Each graph represents an individual ANA. P-values were calculated using a Spearman's rank-order correlation test with significance set to P <0.05.
(TIF)

S8 Fig Map of study site. Figure shows a local map of Uyoma Division, Rarieda District, Kenya, with villages participating in the study highlighted.
(TIF)

S1 Data Raw data. Excel file contains raw data of measured plasma markers in all patients.
(XLSX)
