# Supplementary material for: Aberrant plasma MMP and TIMP dynamics in Schistosoma - Immune reconstitution inflammatory syndrome (IRIS)
Source: PLoS Negl Trop Dis. 2018 Aug 8;12(8):e0006710. doi: 10.1371/journal.pntd.0006710 (PMC6101407; doi:10.1371/journal.pntd.0006710)
Supplement: S1 Table — Prevalence of individual symptoms within selected patients are summarized as number of patients and percentage (n (%)).aTwo unmasking Schisto-IRIS cases are included, whom had 0 eggs pre-ART and had de novo egg production during ART. All paradoxical Schisto-IRIS patients had detectable eggs in stool pre-ART and 4 (44,4%) of them experienced de novo egg production during follow-up. Table includes both new and persistent symptoms. (DOCX) [file pntd.0006710.s001.docx]

**S1 Table. Summary of symptoms in Schisto-IRIS patients and controls**

|  | **Pre-ART** | | |  | **During 3 month follow up** | | |
| --- | --- | --- | --- | --- | --- | --- | --- |
|  | **Schisto-IRIS** _(n=9)_ | **Schisto^+^HIV^+^** _(n=9)_ | **Schisto^-^HIV^+^** _(n=9)_ |  | **Schisto-IRIS** _(n=9)_ | **Schisto^+^HIV^+^** _(n=9)_ | **Schisto^-^HIV^+^** _(n=9)_ |
| Portal vein enlargement | 0 (0%) | 0 (0%) | 0 (0%) |  | 5 (55,6%) | 0 (0%) | 4 (44,4%) |
| Bloody diarrhea | 2 (22.2%) | 1 (11.1%) | 1 (11.1%) |  | 6 (66,7%) | 1 (11,1%) | 1 (11,1%) |
| Hematuria | 1 (11.1%) | 0 (0%) | 0 (0%) |  | 1 (11,1%) | 0 (0%) | 1 (11,1%) |
| Hepatomegaly | 0 (0%) | 2 (22.2%) | 1 (11.1%) |  | 2 (22,2%) | 0 (0%) | 0 (0%) |
| Splenomegaly | 6 (66.7%) | 1 (11.1%) | 5 (55.6%) |  | 4 (44,4%) | 0 (0%) | 0 (0%) |
| Eggs in stool^a^ | 7 (78%) | 9 (100%) | 0 (0%) |  | 6 (67%) | 3 (33%) | 0 (0%) |
| Watery diarrhea | 3 (33.3%) | 3 (33.3%) | 2 (22.2%) |  | 7 (77,8%) | 2 (22,2%) | 2 (22,2%) |
| Fever | 1 (11.1%) | 4 (44.4%) | 4 (44.4%) |  | 6 (66,7%) | 4 (44,4%) | 3 (33,3%) |
| Night sweats | 0 (0%) | 0 (0%) | 1 (11.1%) |  | 2 (22,2%) | 2 (22,2%) | 1 (11,1%) |
| Skin infection | 2 (22.2%) | 1 (11.1%) | 2 (22.2%) |  | 6 (66,7%) | 3 (33,3%) | 2 (22,2%) |
| Skin rash | 1 (11.1%) | 0 (0%) | 1 (11.1%) |  | 0 (0%) | 2 (22,2%) | 2 (22,2%) |
| Swollen Glands | 1 (11.1%) | 0 (0%) | 0 (0%) |  | 1 (11,1%) | 0 (0%) | 0 (0%) |
| Mouth sores | 0 (0%) | 0 (0%) | 1 (11.1%) |  | 1 (11,1%) | 0 (0%) | 1 (11,1%) |
| Chest pains | 1 (11.1%) | 0 (0%) | 2 (22.2%) |  | 2 (22,2%) | 2 (22,2%) | 3 (33,3%) |
| Joint pains | 0 (0%) | 4 (44.4%) | 2 (22.2%) |  | 2 (22,2%) | 3 (33,3%) | 2 (22,2%) |
| Abd. pains | 5 (55.6%) | 6 (66.7%) | 5 (55.6%) |  | 7 (77,8%) | 7 (77,8%) | 6 (66,7%) |
| Abd.discomfort | 4 (44.4%) | 3 (33.3%) | 5 (55.6%) |  | 7 (77,8%) | 6 (66,7%) | 5 (55,6%) |
| Cough | 1 (11.1%) | 1 (11.1%) | 1 (11.1%) |  | 3 (33,3%) | 3 (33,3%) | 1 (11,1%) |
| Headache | 1 (11.1%) | 3 (33.3%) | 3 (33.3%) |  | 6 (66,7%) | 4 (44,4%) | 3 (33,3%) |
| Fatigue | 0 (0%) | 4 (44.4%) | 1 (11.1%) |  | 3 (33,3%) | 5 (55,6%) | 8 (88,9%) |
| Vomiting | 0 (0%) | 2 (22.2%) | 1 (11.1%) |  | 0 (0%) | 2 (22,2%) | 1 (11,1%) |
| Loss of app | 0 (0%) | 3 (33.3%) | 1 (11.1%) |  | 5 (55,6%) | 3 (33,3%) | 4 (44,4%) |
| Weight loss | 7 (77.8%) | 4 (44.4%) | 4 (44.4%) |  | 8 (88,9%) | 7 (77,8%) | 6 (66,7%) |
| Dysuria | 0 (0%) | 0 (0%) | 0 (0%) |  | 1 (11,1%) | 0 (0%) | 0 (0%) |

Prevalence of individual symptoms within selected patients are summarized as number of patients and percentage (n (%)).^a^Two unmasking Schisto-IRIS cases are included, whom had 0 eggs pre-ART and had de novo egg production during ART. All paradoxical Schisto-IRIS patients had detectable eggs in stool pre-ART and 4 (44,4%) of them experienced de novo egg production during follow-up. Table includes both new and persistent symptoms.
