# Supplementary figures and images for: Aberrant plasma MMP and TIMP dynamics in Schistosoma - Immune reconstitution inflammatory syndrome (IRIS)
Source: PLoS Negl Trop Dis. 2018 Aug 8;12(8):e0006710. doi: 10.1371/journal.pntd.0006710 (PMC6101407; doi:10.1371/journal.pntd.0006710)

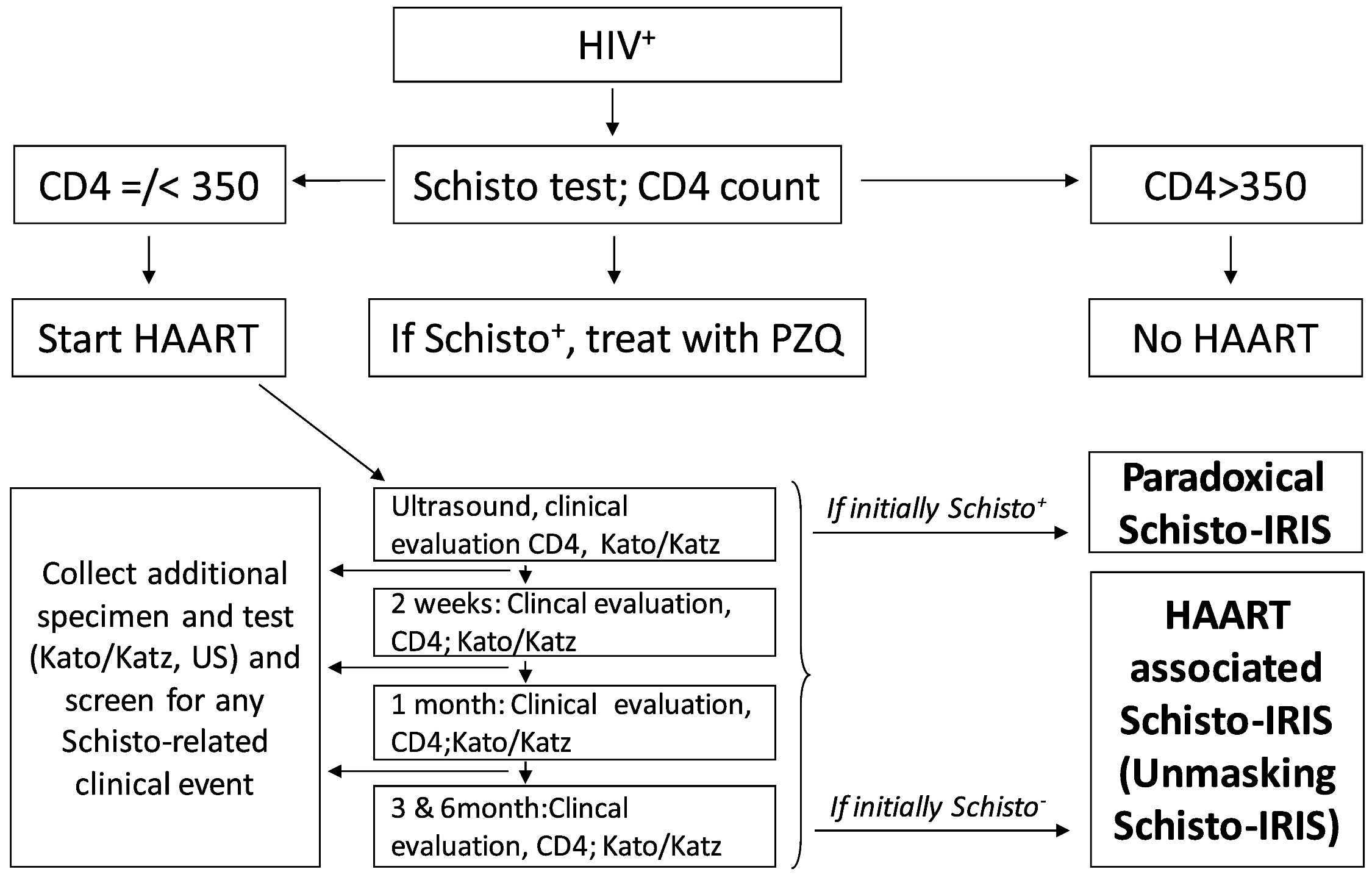

Supplement: S1 Fig — Figure shows follow-up of HIV patients during ART, with sequential testing for clinical parameters to diagnose schistosomiasis and IRIS. (TIF) [file pntd.0006710.s002.tif]

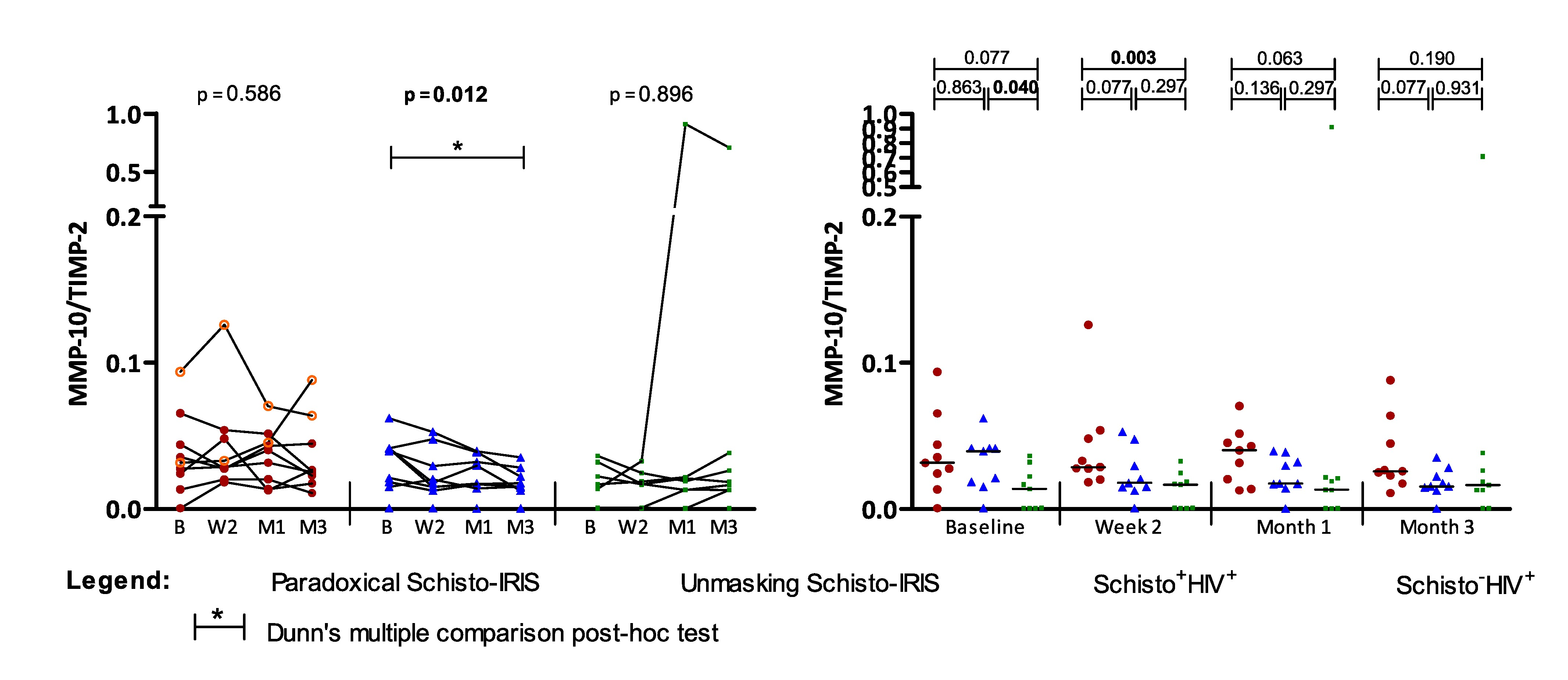

Supplement: S2 Fig — Figure shows (A) time analysis of MMP-10/TIMP-2 ratio in 3 patient groups using a Friedman test (p-values shown in graphs), with Dunn’s multiple comparison post-hoc tests to indicate differences between time points when applicable (indicated by horizontal bars with an asterisk). (B) comparison of MMP-10/TIMP-2 ratio between groups for each time point using a Mann-whitney U test (p-values and horizontal bars). (TIF) [file pntd.0006710.s003.tif]

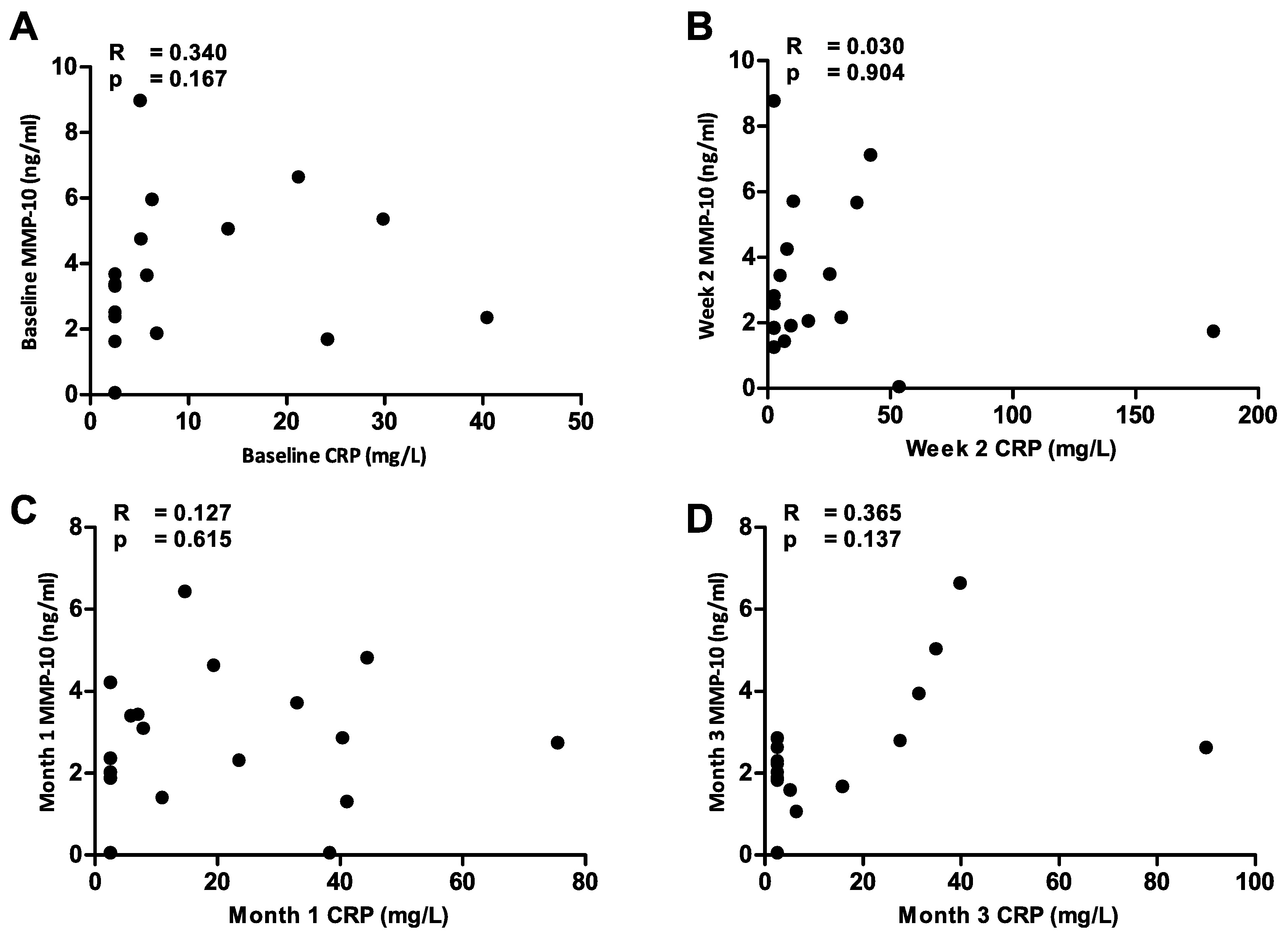

Supplement: S3 Fig — Figure shows a correlation within pooled Schisto-IRIS & Schisto+HIV+ patients between plasma CRP and MMP-10 levels at (A) baseline, (B) week 2, (C) month 1, and (D) month 3. P-values were calculated using a Spearman's rank-order correlation test with significance set to P <0.05. (TIF) [file pntd.0006710.s004.tif]

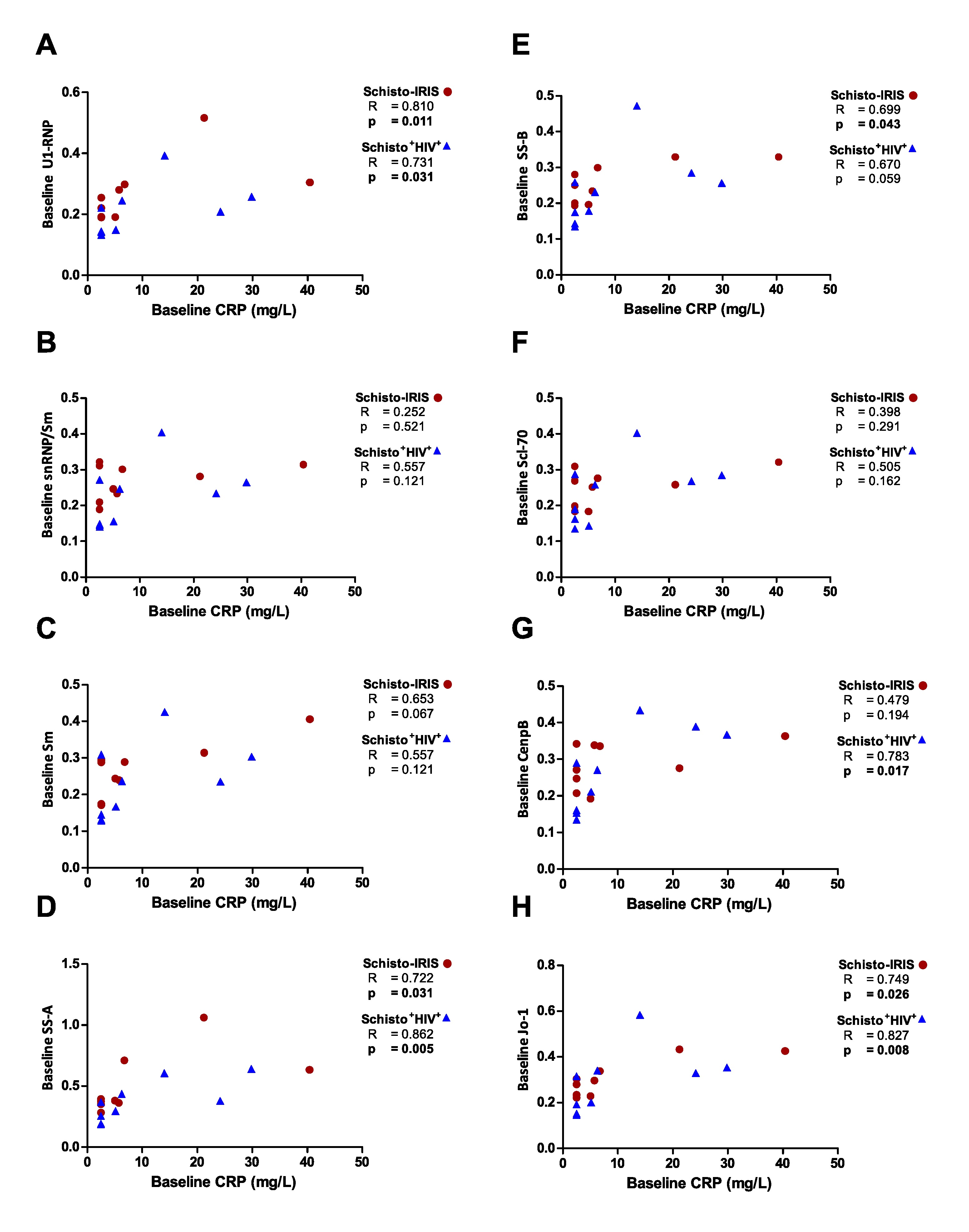

Supplement: S4 Fig — Figure shows a correlation in Schisto-IRIS (red circles) and Schisto+HIV+ (blue triangles) patients between baseline values for CRP and (A) U1-RNP, (B) snRNP/Sm, (C) Sm, (D) SS-A, (E) SS-B, (F) Scl-70, (G) CenpB, and (H) Jo-1. Each graph represents an individual ANA. P-values were calculated using a Spearman's rank-order correlation test with significance set to P <0.05. (TIF) [file pntd.0006710.s005.tif]

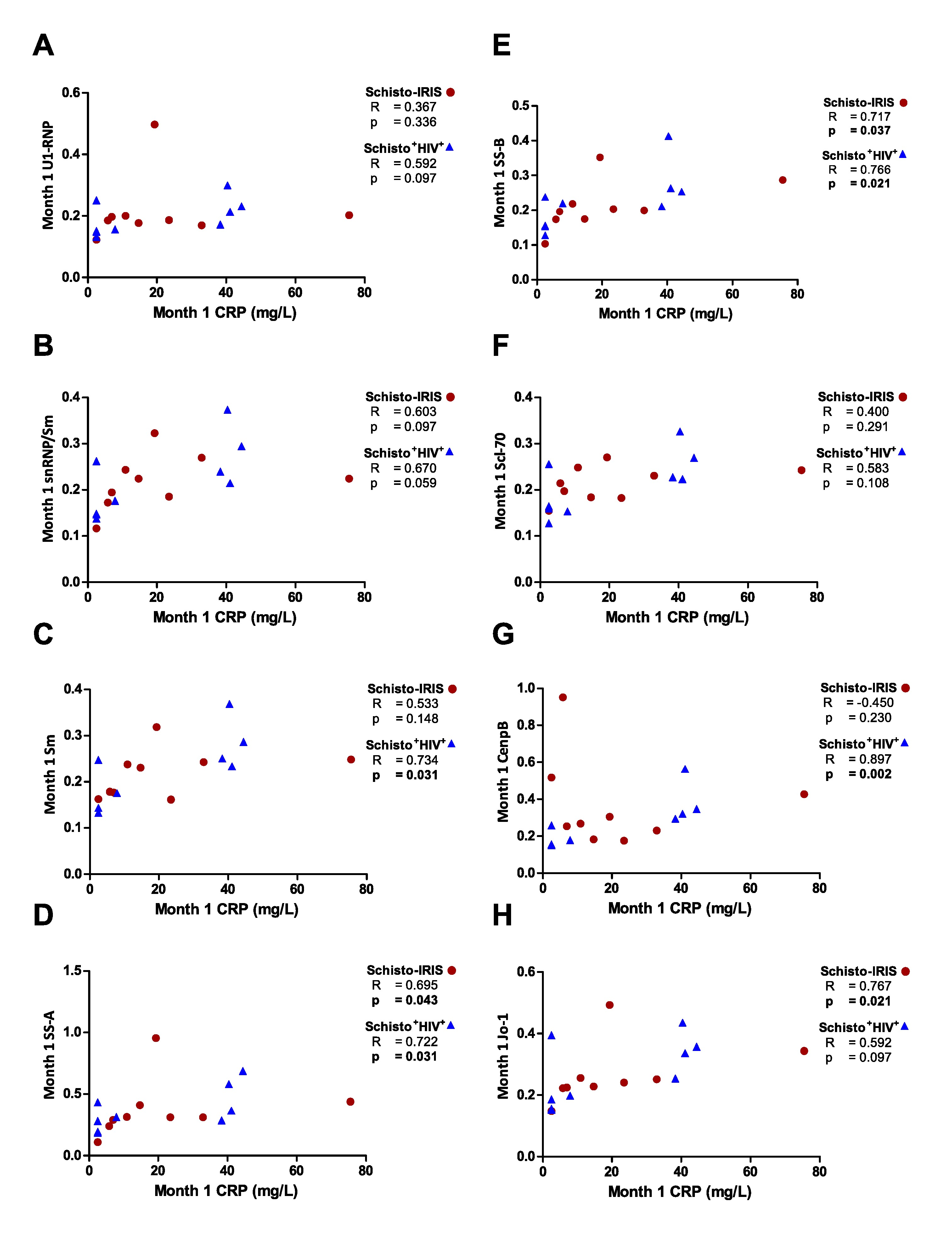

Supplement: S5 Fig — Figure shows a correlation in Schisto-IRIS (red circles) and Schisto+HIV+ (blue triangles) patients between month 1 values for CRP and (A) U1-RNP, (B) snRNP/Sm, (C) Sm, (D) SS-A, (E) SS-B, (F) Scl-70, (G) CenpB, and (H) Jo-1. Each graph represents an individual ANA. P-values were calculated using a Spearman's rank-order correlation test with significance set to P <0.05. (TIF) [file pntd.0006710.s006.tif]

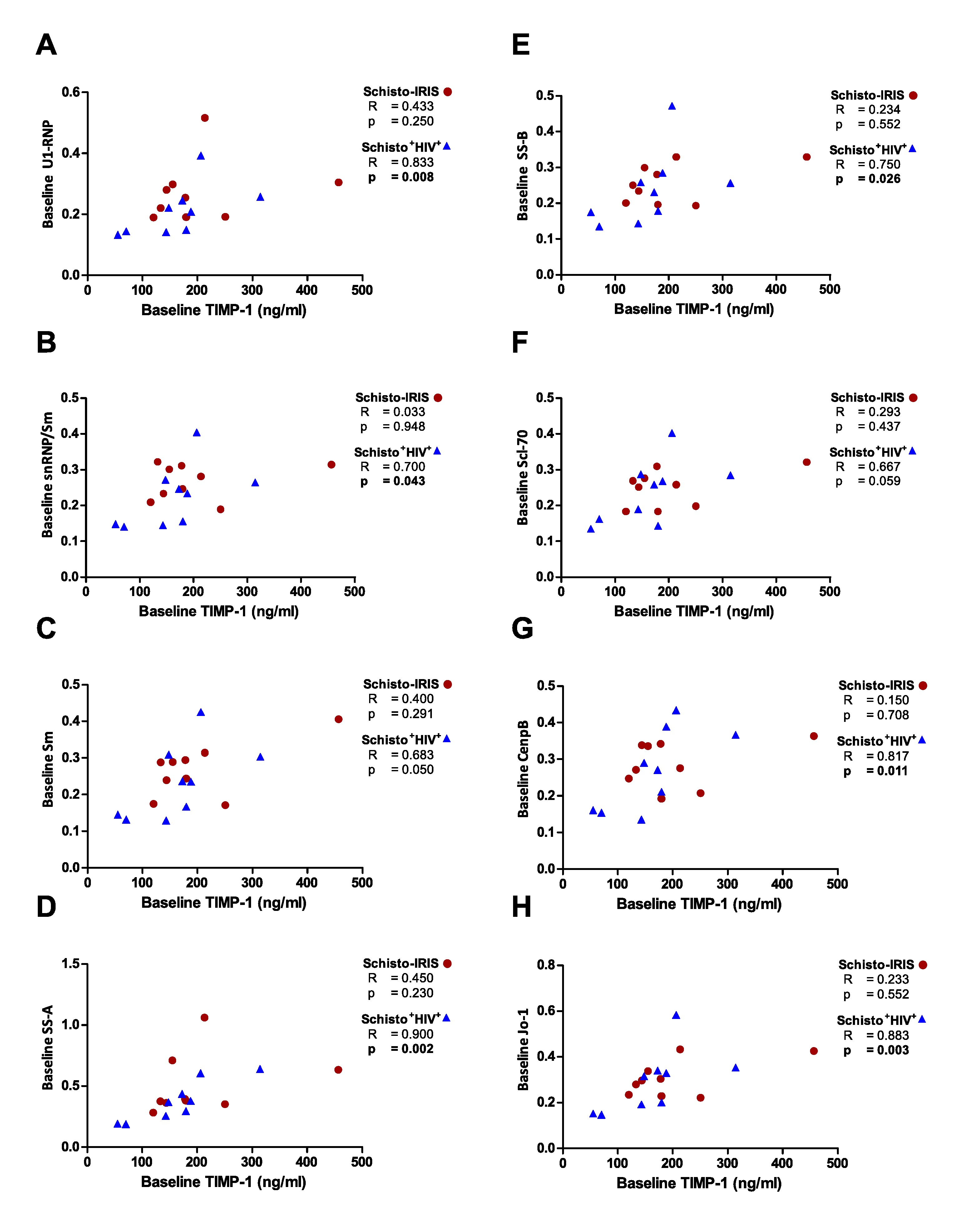

Supplement: S6 Fig — Figure shows a correlation in Schisto-IRIS (red circles) and Schisto+HIV+ (blue triangles) patients between baseline values for TIMP-1 and (A) U1-RNP, (B) snRNP/Sm, (C) Sm, (D) SS-A, (E) SS-B, (F) Scl-70, (G) CenpB, and (H) Jo-1. Each graph represents an individual ANA. P-values were calculated using a Spearman's rank-order correlation test with significance set to P <0.05. (TIF) [file pntd.0006710.s007.tif]

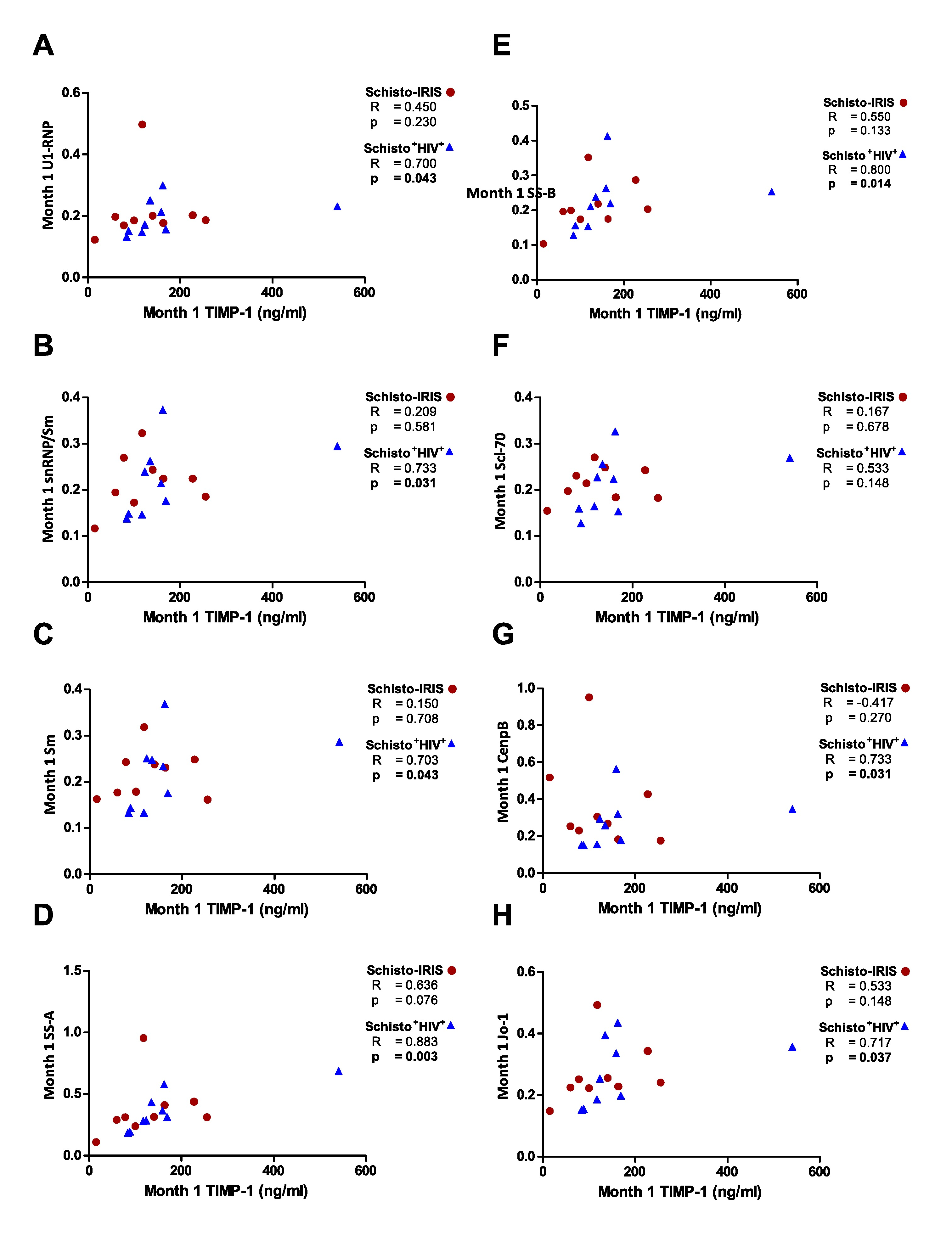

Supplement: S7 Fig — Figure shows a correlation in Schisto-IRIS (red circles) and Schisto+HIV+ (blue triangles) patients between month 1 values for TIMP-1 and (A) U1-RNP, (B) snRNP/Sm, (C) Sm, (D) SS-A, (E) SS-B, (F) Scl-70, (G) CenpB, and (H) Jo-1. Each graph represents an individual ANA. P-values were calculated using a Spearman's rank-order correlation test with significance set to P <0.05. (TIF) [file pntd.0006710.s008.tif]

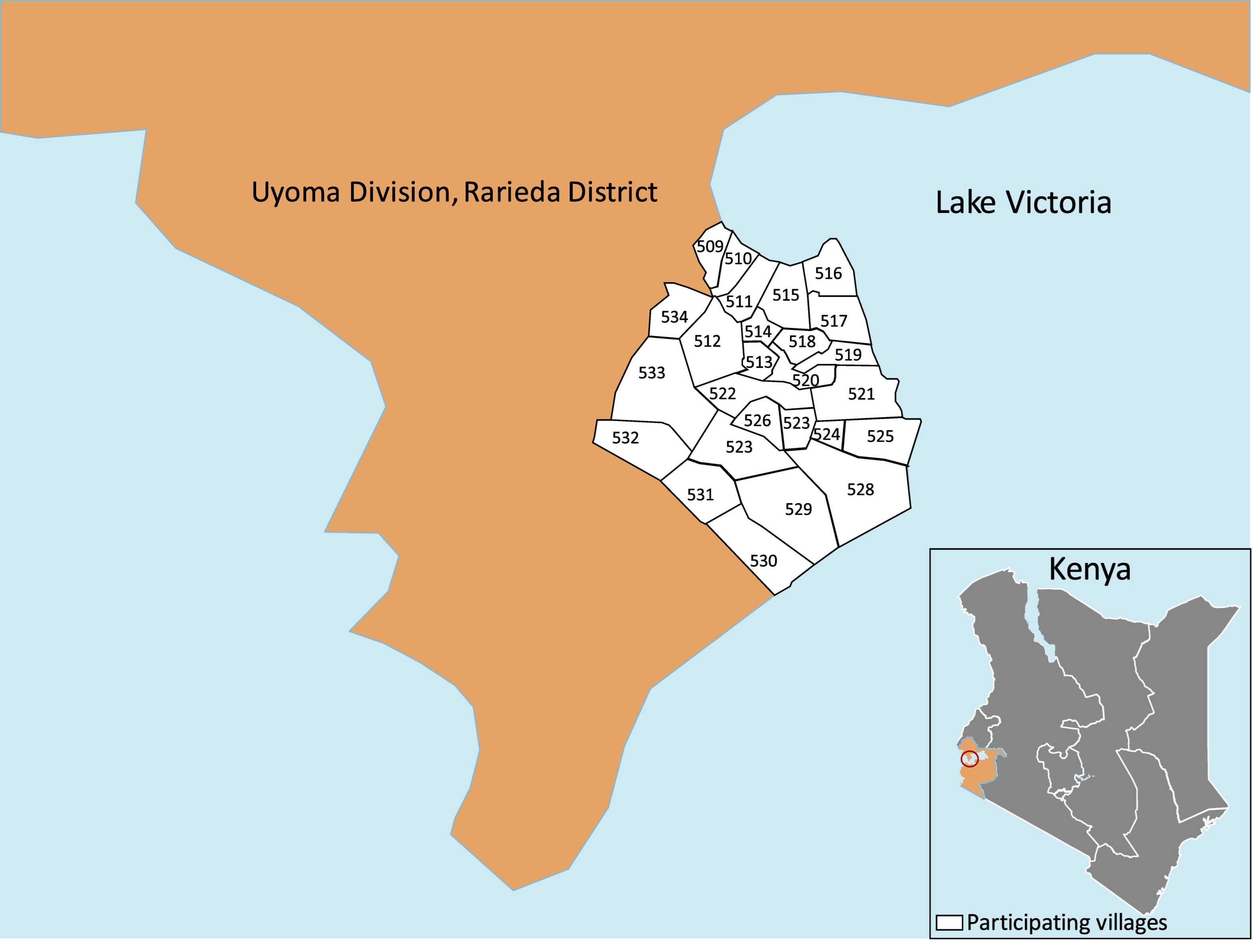

Supplement: S8 Fig — Figure shows a local map of Uyoma Division, Rarieda District, Kenya, with villages participating in the study highlighted. (TIF) [file pntd.0006710.s009.tif]
